# Effects of a wide range of dietary forage-to-concentrate ratios on nutrient utilization and hepatic transcriptional profiles in limit-fed Holstein heifers

**DOI:** 10.1186/s12864-018-4529-9

**Published:** 2018-02-17

**Authors:** Haitao Shi, Jun Zhang, Shengli Li, Shoukun Ji, Zhijun Cao, Hongtao Zhang, Yajing Wang

**Affiliations:** 1 State Key Laboratory of Animal Nutrition, Beijing Engineering Technology Research Center of Raw Milk Quality and Safety Control, College of Animal Science and Technology, China Agricultural University, No.2 Yuanmingyuan West Road, Haidian, Beijing, 100193 People’s Republic of China; 2 Department of Animal and Poultry Science, University of Saskatchewan, Saskatoon, SK S7N 5A8 Canada

**Keywords:** Energy utilization, Lipid metabolism, Forage level, Liver, Heifer, RNA-Seq

## Abstract

**Background:**

Improving the efficiency of animal production is a relentless pursuit of ruminant producers. Energy utilization and partition can be affected by dietary composition and nutrient availability. Furthermore, the liver is the central metabolic intersection in cattle. However, the specific metabolic changes in the liver under conditions of limit-feeding remain unclear and require further study. The present study aimed to elucidate the effects of a wide range of dietary forage:concentrate ratios (F:C) on energy utilization, and identify potential changes in molecular metabolism by analyzing hepatic transcriptional profiles. Twenty-four half-sib Holstein heifers were fed four F:C diets (20:80, 40:60, 60:40, and 80:20 on a dry matter basis), with similar intake levels of metabolizable energy (ME) and crude protein. Liver biopsy samples were obtained and RNA sequencing was conducted to identify the hepatic transcriptomic changes. Moreover, the ruminal fermentation profiles, growth characteristics, and levels of metabolites in the liver and plasma of the heifers were monitored.

**Results:**

The proportion of acetate showed a linear increase (*P* < 0.01) with increasing dietary forage levels, whereas the proportion of propionate showed a linear decline (*P* ≤ 0.01). Lower levels of average daily gain and feed efficiency (*P* < 0.01) were observed in heifers fed high levels of forage, with a significant linear response. Using the Short Time-series Expression Miner software package, the expression trends of significant differentially expressed genes (DEGs) were generally divided into 20 clusters, according to their dynamic expression patterns. Functional classification analysis showed that lipid metabolism (particularly cholesterol and steroid metabolism which were in line with the cholesterol content in the liver and plasma) was significantly increased with increasing dietary forage levels and slightly reduced by the 80% forage diet. Nine DEGs were enriched in the related pathways, namely *HMGCS1*, *HMGCR*, *MSMO1*, *MVK*, *MVD*, *IDI1*, *FDPS*, *LSS*, and *DHCR7*.

**Conclusions:**

The ruminal fermentation and feed efficiency results suggest that different mechanisms of energy utilization might occur in heifers fed different F:C diets with similar levels of ME intake. Increased cholesterol synthesis from acetate might be responsible for the reduced efficiency of energy utilization in heifers fed high-forage diets.

**Electronic supplementary material:**

The online version of this article (10.1186/s12864-018-4529-9) contains supplementary material, which is available to authorized users.

## Background

Ruminants are unique in their ability to convert large quantities of plant fiber into high-quality products, like meat and milk, for human consumption. With rapid growth in the human population, there is increasing demand for livestock products. Improving the efficiency of livestock production is a crucial requirement to meet this challenge and minimize any potential environmental impact [1]. Ruminants rely largely on the ruminal fermentation of feedstuffs by microorganisms to obtain nutrients, like volatile fatty acids (VFAs), proteins, and vitamins for maintenance, growth, and production [2]. Energy metabolism in heifers plays an important role in regulating feed efficiency, which ultimately affects growth, production performance, as well as the conception rate [3, 4].

Previous studies report that nutrient levels have a significant effect on metabolic activities and rate, and dietary forage:concentrate ratios (F:C) might affect visceral organ mass and/or metabolism, resulting in altered levels of available energy for weight gain and/or efficiency of gain [5–7]. Recently, limit-feeding of low-forage diets has been proposed as an effective method to improve energy efficiency in heifers, and had been explored by several researchers [8–10]. In most cases, limit-feeding means that heifers are fed low-forage diets to meet, but not exceed energy requirements for an appropriate level of average daily gain (ADG) [11, 12]. However, details of the associated physiological and metabolic mechanisms and changes remain to be further characterized.

The liver is the central metabolic intersection between portal-drained viscera (PDV) and the rest of body, and it accounts for 17%–25% of whole-body oxygen uptake or energy loss as heat, and only 1%–1.5% of total body weight [6, 13]. As a central organ responsible for intermediary metabolism within the body, it is not surprising that the metabolic activities of liver would be affected by dietary composition and nutrient intake [14]. Coordination of the flux and inter-conversion of nutrients and metabolites in the liver might thus be altered by changes in the diet. The associated processes may be controlled by various means, including alterations of gene expression, enzyme activity, and the resultant nutrient fluxes that optimize liver function and nutrient inter-conversion [14]. Therefore, increased understanding of how the F:C influences global hepatic gene expression profiles may lead to improved approaches to enhancing the efficiency of energy utilization in ruminant production systems. Furthermore, only two or three forage levels (76% vs. 24%; 90% vs. 45%; and 75% vs. 25%) have been used in previous studies [6, 8, 12]. These designs however, do not provide a large enough range with which models can be developed to predict physiological variation, thereby leading to a better understanding of energy utilization in heifers.

High-throughput RNA sequencing (RNA-Seq) is rapidly emerging as a favored approach for transcriptome-oriented studies, and can be used to analyze changes in gene expression across the entire transcriptome [15, 16]. With sensitive, unbiased detection of all expressed genes, this method has been successfully used in the identification of potential transcriptional mechanisms and components in bovine species subjected to different phenotypic and physiological changes. These include liver transcripts associated with dietary restriction [14], negative energy balance [17], and different levels of feed efficiency [18] in cattle. Therefore, RNA-Seq based transcriptomic profiling was used in the present study to identify the effects and underlying mechanism of four F:C diets on hepatic gene expression profiles in heifers with an equal intake of metabolizable energy (ME). Moreover, the changes in ruminal fermentation, growth characteristics, and plasma metabolites were also analyzed.

## Methods

### Animals, experimental design, management, and diets

Animal care for the experiment complied with the Regulations for the Administration of Affairs Concerning Experimental Animals, National Committee of Science and Technology of China (14 November 1988) and Instructive Notions with Respect to Caring for Experimental Animals, Ministry of Science and Technology of China (13 September 2006). The animal procedures were approved by the Ethical Committee of the College of Animal Science and Technology of China Agricultural University.

Twenty-four half-sib Holstein heifers (8–10 months of age and 253 ± 29 kg in body weight [BW] at the beginning of the study) from Beijing Sanyuan Lvhe Dairy Group (Beijing, China) were used for a 4-week feeding trial following a pre-experimental period of 4 weeks. Half-sib heifers were used to minimize the differences in genotype between individual animals. The animals were privately owned by the Beijing Sanyuan Lvhe Dairy Group and permissions for the animals to be used were obtained prior to initiation of the study. During the pre-experimental period, all heifers were fed an adaptation diet containing 50% corn silage and 50% concentrate (on a dry matter (DM) basis; Additional file 1: Table S1). At the end of pre-experimental period, heifers were blocked by BW in a randomized complete block design, and assigned to four different F:C diets (20:80, 40:60, 60:40, and 80:20 on a DM basis, namely the S20, S40, S60, and S80 groups, respectively; Additional file 1: Table S1), with corn silage as the sole forage source. All diets were formulated to meet or exceed the NRC (2001) nutrient recommendations [19], and were provided as a total mixed ration (TMR) at levels calculated to provide a similar intake of ME and crude protein (CP).

Heifers were individually fed a TMR twice daily at 12-h intervals (0700 and 1900 h). Water was available ad libitum. Dry matter intake (DMI) for each heifer was recorded daily. Samples of individual feedstuffs and TMR were collected daily and stored at − 20 °C. At the end of each week, the daily samples were pooled and subsamples were taken for chemical analysis.

### Ruminal sample collection, measurements, and growth characteristics

At the end of the feeding period (day 28), rumen contents (100 mL/heifer) were collected using an oral stomach tube approximately 4 h after morning feeding, according to previously reported procedures [10]. Ruminal fluid that had been filtered (through four layers of cheesecloth) was stored at − 20 °C, before the concentrations of NH_3_-N and VFAs were analyzed according to previously reported procedures [10, 20].

Body weight was measured on two consecutive days each week (approximately 1 h before morning feeding) and the mean values were used to adjust the TMR offered, and account for day-to-day variations [21]. The gut fill, ADG (after removing the effects of gut fill), and feed efficiency (FE, after removing effects of gut fill, FE = ADG/DMI) were calculated, using prediction equations described by Williams et al. [22].

### Blood and liver sample collection, and metabolite measurements

Blood samples were collected about 6 h after morning feeding from the jugular vein into 5-mL lithium heparin vacuum tubes (Hebei Xinle Medical, Shijiazhuang, China) on day 28 of the experimental period. All tubes were centrifuged at 3000 *g* at 4 °C for 15 min to obtain plasma, which was stored at − 20 °C until further analysis. The plasma activity/concentration levels of alanine aminotransferase (ALT), alkaline phosphatase, aspartate aminotransferase, β-hydroxybutyric acid, blood urea nitrogen, glucose, lactate dehydrogenase, low-density lipoprotein cholesterol (LDL-C), total amino acids, total bilirubin, total cholesterol (TC), total triglyceride (TG), total protein, and non-esterified fatty acids were analyzed, using a Clinical HITACHI (7160) Automatic Analyzer (Hitachi Limited, Tokyo, Japan) with kits supplied by an instrumentation laboratory. Plasma concentration of high-density lipoprotein cholesterol (HDL-C) was measured using an immunoinhibition method (kits from Beijing Beijian Xinchuangyuan Biotechnology Ltd.). Plasma levels of very low-density lipoprotein cholesterol (VLDL-C) were measured, using enzyme-linked immunosorbent assay (ELISA) kits (Shanghai Huole Biotechnology Ltd.), according to the manufacturer’s instructions.

Liver biopsies from each heifer were obtained immediately after blood sampling by percutaneous needle biopsy, as previously described [23, 24]. Briefly, the skin was shaved and disinfected and the area through the skin and body wall was anesthetized with 2% lignocaine (Shanghai Harvest Pharmaceutical Co., Ltd., Shanghai, China). A stab incision was made through the skin in the right 11th intercostals space at the level of the greater trochanter, through which a biopsy needle was inserted into the liver and around 500–1000 mg liver tissue was collected. All biopsies were then rinsed in saline and immediately frozen in liquid N until further analysis. For metabolite analysis, 0.9 mL absolute ethyl alcohol was added to a 100-mg liver sample. The mixture was homogenized with a microelectric homogenizer (Kontes) for 30 s, and then centrifuged at 2000 *g* at 4 °C for 20 min to obtain a suspension. The TC and TG concentrations in the liver were analyzed using a commercially available enzymatic kit (Nanjing Jiancheng Bioengineering Institute, Nanjing, China).

### Liver RNA isolation, sequencing, and sequence data processing

Total RNA from liver biopsies was isolated using the QIAzol Lysis Reagent (Qiagen, Hilden, Germany), followed by purification on the miRNeasy mini column (miRNeasy Mini Kit, Qiagen, Hilden, Germany), according to the manufacturer’s protocols. The RNA quality and purity were checked using an ND-100 NanoDrop spectrophotometer (NanoDrop Technologies, Wilmington, DE, USA). Any RNA integrity number (RIN) > 8.0 was deemed to be of sufficiently high quality. RNA degradation and contamination were monitored on 1.0% agarose gels.

Briefly, 2.5 μg of total RNA was enriched by Poly-A using the NEBNext Poly (A) mRNA Magnetic Isolation Module (NEB, E7490S). A transcriptome library was constructed for sequencing, according to the protocols of the NEBNext Ultra RNA Library Prep Kit for Illumina (NEB, E7530S) and NEBNext Multiplex Oligos for Illumina (NEB, E7500S). The prepared library was quantified using the KAPA Library Quantification Kit-Illumina GA Universal (Kapa, KK4824), and subjected to 1.8% agarose gel electrophoresis to be validated for quality. The library products were sequenced via an Illumina HiSeq 2500 sequencer. Both library building and sequencing were performed by CapitalBio Corporation (Beijing, China).

Raw reads were cleaned by removing adapter and primer sequences, reads containing < 10 nt, and low-quality reads (more than half of the reads with a phred base quality score of < 5) from the raw data. Ribosome RNA sequences were filtered from the raw fragments. All downstream analyses were based on the clean reads.

An index of the reference genome was built using Bowtie v1.1.2, and paired-end clean reads for each individual were aligned to the bovine reference genome (UMD3.1) using TopHat v2.1.0, and then assembled using the Cufflinks package [14, 25]. The differentially expressed genes (DEGs) were detected by Cuffdiff, which is included in the Cufflinks package. The commonly used fragments per kilobase of exon per million fragments mapped (FPKM) in pair-end sequencing experiments was incorporated in two normalization steps; i.e. the number of fragments were normalized by the transcript’s length and total yield of the fragments to ensure accurate quantification of the gene’s expression [25]. Genes with a false discovery rate (FDR, *q*-value) ≤ 0.05 and a fold change (FC) greater than 1.5 were considered DEGs.

### Gene ontology and pathway analysis

The overall DEGs (*q*-value ≤0.05 and FC ≥ 1.5) were analyzed for enrichment of Gene Ontology (GO) terms (biological process (BP), molecular function (MF) and cellular component (CC)) and Kyoto Encyclopedia of Genes and Genomes database (KEGG) pathways using DAVID Bioinformatics Resources [26]. For GO analysis, values of *P* ≤ 0.05 identified differentially enriched terms, and genes with a *q*-value < 0.05 among the DEGs were considered significantly enriched. Significant differentially enriched KEGG pathways were considered as those with a value of *P* ≤ 0.05.

### Short time-series expression miner (STEM) analysis

Assignments of DEGs to temporal expression profiles and detection of statistically enriched gene families within each profile was conducted using STEM v1.3.8 [27], with the maximum number of model profiles set to 20, and maximum unit change in model profiles between time points set to 2. The expression data (FPKM value) were normalized to 0 [log2(S20/S20)], log2(S40/S20), log2(S60/S20), and log2(S80/S20) when input to STEM. Each gene was assigned to the closest profile using a Pearson correlation-based distance metric. To determine the significance level for a given transcriptome profile, a permutation-based test was used to quantify the expected number of genes that would be assigned to each profile [28]. The *P*-value derived from STEM analysis was corrected for multiple hypothesis testing, using a *q*-value < 0.05. To understand further the biological functions of DEGs clustered in STEM, GO and KEGG analyses were also performed using DAVID Bioinformatics Resources [26].

### The quantitative real-time PCR (qRT-PCR) protocol

To validate the repeatability and reproducibility of gene expression data obtained by RNA sequencing in the Holstein heifers, quantitative real-time PCR was carried out on five randomly selected DEGs (*DHCR7*, *LSS*, *HSPB1*, *ATF4*, and *GOT1*) using the total RNA used for RNA-Seq. The PrimeScript™ RT Master Mix (Takara, Dalin, China) was used to synthesize first-strand cDNA according to the manufacturer’s protocol. Primers were designed by Primer5 (http://www.premierbiosoft.com/primerdesign/index.html), and the sequences are presented in Additional file 1: Table S2. The qRT-PCR was carried out in triplicate with a final volume of 10 μL using the SYBR® Premix Ex Taq™ II (Takara, Dalin, China) on the QuantStudio™ 6 Flex system (Life Technologies, USA), following the manufacturer’s protocol. The relative expression of target genes was normalized to the expression of *GAPDH* and *β-actin*, and calculated using the 2^-ΔΔCt^ method [29–31].

### Statistical analyses

Data for ruminal fermentation parameters, growth characteristics, liver and plasma metabolites, and qRT-PCR were analyzed in a randomized complete block design using the PROC MIXED procedure of the SAS software (SAS Institute Inc., Cary, NC, USA). The model included treatment and block as fixed effects. Results were reported as least squares means, and compared using the Tukey’s test. Contrasts were used to test the linear, quadratic, and cubic changes effected by increasing levels of dietary forage. Significant differences were declared at *P* ≤ 0.05, and trends were reported at 0.05 < *P* < 0.10.

## Results

### Ruminal fermentation parameters and growth characteristics

The concentration of NH_3_-N showed a quadratic decline (*P* < 0.01) in the S60 group, which had the lowest value among all treatments. Ruminal proportions of acetate, and the ratio of acetate:propionate (A:P) showed a linear increase (*P* < 0.01) with increasing levels of forage in the diets (Table 1). The proportions of propionate and butyrate showed a linear decline (*P* ≤ 0.01) with increasing dietary forage levels. The proportions of isobutyrate showed a quadratic increase (*P* = 0.02), with the S60 group yielding the highest value with increasing dietary forage levels. The proportions of isovalerate were cubically influenced (*P* = 0.04) with increasing dietary forage levels.

**Table 1:** Effects of dietary forage levels on ruminal fermentation in Holstein heifers

Items	Forage levels (% of diet DM)				SEM^f^	*P*-value			
	20	40	60	80		Treatment	Linear	Quadratic	Cubic
NH_3_-N, mg/dL	8.27^a^	3.93^b^	2.22^b^	2.40^b^	0.583	<0.01	<0.01	<0.01	0.78
TVFAs^e^, m*M*	94.64	92.49	89.53	86.10	1.806	0.43	0.11	0.87	0.98
VFAs, molar % of TVFAs									
Acetate	53.85^c^	58.04^bc^	62.48^ab^	65.97^a^	1.135	<0.01	<0.01	0.81	0.85
Propionate	25.44^a^	23.31^ab^	22.10^ab^	20.99^b^	0.624	0.04	0.01	0.63	0.86
Butyrate	16.91^a^	12.42^b^	11.05^b^	9.69^b^	0.688	<0.01	<0.01	0.09	0.43
Valerate	0.56	1.08	0.58	0.52	0.100	0.11	0.42	0.11	0.08
Isobutyrate	0.93	2.16	2.29	1.24	0.226	0.10	0.59	0.02	0.97
Isovalerate	2.27	2.99	1.49	1.59	0.223	0.05	0.06	0.43	0.04
A:P^g^	2.16^b^	2.53^ab^	2.89^ab^	3.18^a^	0.115	<0.01	<0.01	0.81	0.96

(*n* = 6)

^a–c^Means within a row with different superscripts differ significantly (*P* < 0.05)

^e^TVFAs total volatile fatty acids

^f^SEM standard error of the mean

^g^A:P acetate:propionate

By the end of the experiment, no significant differences were found in BW among the treatments (Table 2). The final gut fill was calculated and showed a linear increase (*P* < 0.01) with increasing dietary forage levels, whereas the ADG and FE showed a linear decline (*P* < 0.01).

**Table 2:** Effects of dietary forage levels on growth characteristics

Items^e^	Forage levels (% of diet DM)				SEM^f^	*P*-value			
	20	40	60	80		Treatment	Linear	Quadratic	Cubic
Initial BW, kg	262.5	263.8	263.7	262.3	6.096	0.96			
Initial gut fill^g^, kg	38.12	38.81	39.22	39.43	0.448	0.22			
Final BW, kg	284.2	287.0	288.7	290.5	6.330	0.40	0.10	0.82	0.91
Final gut fill^g^, kg	29.89^d^	38.83^c^	47.80^b^	56.91^a^	0.645	< 0.01	< 0.01	0.90	0.97
ADG^g^, kg/d	1.07^a^	0.83^b^	0.59^c^	0.38^c^	0.051	< 0.01	< 0.01	0.72	0.88
FE^g^	0.24^a^	0.18^b^	0.12^c^	0.07^d^	0.009	< 0.01	< 0.01	0.32	0.97

(*n* = 6)

^a–d^Means within a row with different superscripts differ significantly (*P* < 0.05)

^e^*BW* body weight, *ADG* average daily gain, *FE* feed efficiency; *DM* dry matter

^f^*SEM* standard error of the mean

^g^ADG = (final BW − final gut fill) − (initial BW − initial gut fill); FE = ADG/DMI, calculated according to Williams et al. [22]

### Liver and plasma metabolites

As shown in Table 3, liver concentrations of TC and TG were cubically changed (*P* ≤ 0.034) with increasing dietary forage levels; the S60 group yielded the highest values. Plasma concentration of LDL-C showed a linear increase (*P* = 0.001) with increasing dietary forage levels, whereas the concentration of urea showed a linear decline (*P* < 0.001). Plasma concentration of VLDL-C showed a linear (*P* < 0.001) and cubic (*P* < 0.001) response with increasing dietary forage levels; whereas that of TC tended towards a linear increase (*P* = 0.070). Other measured plasma metabolites showed no significant differences among treatments.

**Table 3:** Influence of dietary forage levels on liver and plasma metabolites in Holstein heifers

Items^c^	Forage levels (% of diet DM)				SEM^d^	*P*-value			
	20	40	60	80		Treatment	Linear	Quadratic	Cubic
Liver									
TC, mg/g of liver tissue	1.39^ab^	1.40^ab^	2.53^a^	1.19^b^	0.170	0.029	0.710	0.056	0.026
TG, μmol/g of liver tissue	3.67	3.99	6.72	3.83	0.397	0.027	0.369	0.056	0.034
Plasma									
Total protein, g/L	71.71	71.32	71.57	71.60	0.961	0.993	0.989	0.831	0.843
Glucose, mmol/L	4.54	4.53	4.40	4.49	0.126	0.856	0.624	0.711	0.549
Lactate Dehydrogenase, U/L	996.43	944.55	934.24	951.20	54.359	0.857	0.557	0.536	0.954
Aspartate Aminotransferase, U/L	68.06	71.18	65.03	73.67	4.084	0.490	0.567	0.510	0.207
ALT, U/L	36.78^b^	40.06^b^	44.07^ab^	53.60^a^	2.312	0.007	< 0.001	0.197	0.650
TC, mmol/L	2.46	2.74	2.92	2.81	0.141	0.169	0.070	0.188	0.773
TG, mmol/L	0.37	0.31	0.44	0.39	0.051	0.407	0.451	0.904	0.136
Alkaline Phosphatase, mmol/L	141.94	129.10	139.79	157.86	13.350	0.518	0.343	0.265	0.790
Urea, mmol/L	3.33^a^	3.19^a^	3.09^ab^	2.55^b^	0.113	0.001	< 0.001	0.097	0.362
*β*-hydroxybutyric acid, mmol/L	0.13	0.12	0.11	0.12	0.005	0.204	0.057	0.493	0.520
Total amino acids, mmol/L	3.70	3.91	4.57	3.30	0.473	0.324	0.803	0.140	0.275
Nonesterified Fatty Acid, mmol/L	0.47	0.46	0.45	0.44	0.014	0.531	0.162	0.783	0.820
Total Bilirubin, μmol/L	12.02	11.83	11.22	12.52	0.581	0.486	0.739	0.222	0.380
VLDL-C, mmol/L	0.53	0.75	0.58	0.94	0.060	0.001	0.002	0.259	0.003
LDL-C, mmol/L	0.58	0.58	0.96	0.98	0.085	0.004	0.001	0.893	0.077
HDL-C, mmol/L	1.63	1.71	1.63	1.52	0.094	0.579	0.348	0.326	0.801

(*n* = 6)

^a–b^Means within a row with different superscripts differ significantly (*P* < 0.05)

^c^*ALT* alanine aminotransferase, *TC* total cholesterol, *TG* total triglycerides, *VLDL-C* very low-density lipoprotein cholesterol, *LDL-C* low-density lipoprotein cholesterol, *HDL-C* high-density lipoprotein cholesterol

^d^*SEM* standard error of the mean

### Sequencing and characterization of the bovine liver transcriptome

As shown in Table 4, the cDNA libraries of 24 liver biopsy samples from heifers fed the four experimental diets were sequenced (125 bp paired-end strategy), and in total 39,273,652 to 55,427,092 paired-end reads were obtained. After filtering low-quality reads and adapters from raw fragments, 38,637,820 to 54,562,265 high-quality clean reads in each sample were obtained. Among the high-quality reads, 91.88%–93.87% had base-call quality at Q30 (proportion of bases with a phred base-quality score > 30); and 99.69%–99.85% at Q20 (proportion of bases with a phred base-quality score > 20). Alignment of sequence reads against the bovine genome UMD3.1 yielded 86.87%–90.60% of aligned reads across the 24 samples. Among these, 81.52%–83.88% were uniquely aligned reads that were used for further analysis, to verify the reliability of the results.

**Table 4:** Statistics for RNA-Seq reads from liver biopsy, and alignment information with Tophat^a^ (group means)

Mapping summary	Forage levels (% of diet DM)			
	20	40	60	80
Total reads (raw reads)	45,397,012	44,701,037	43,415,394	45,897,090
Clean reads	44,679,767	43,983,934	42,714,824	45,155,970
Statistic for clean reads: Q20^b^	99.79%	99.83%	99.83%	99.83%
Statistic for clean reads: Q30^c^	92.88%	92.94%	92.80%	92.80%
Statistic for clean reads: GC content	44.17%	45.00%	44.00%	44.00%
Statistic for clean reads: Seq-Dupl-level^d^	85.18%	83.91%	84.30%	82.52%
Mapping rate	89.72%	90.20%	90.03%	89.97%
Total mapped reads	40,109,810	39,668,475	38,458,347	40,641,575
Multiple mapped reads	2,938,690	2,958,931	2,849,620	3,070,914
Unique mapped reads	37,171,120	36,709,544	35,608,727	37,570,661
Junction mapped reads	19,339,604	18,902,568	18,610,456	19,784,290
Statistic for clean reads: error rate	0.29%	0.30%	0.30%	0.31%

^a^Bovine genome UMD3.1 was used for the alignment

^b^Q20, the proportion of bases with a phred base-quality score > 20; i.e., the proportion of read bases with an error rate less than 1%

^c^Q30, the proportion of bases with a phred base-quality score > 30; i.e., the proportion of read bases with an error rate less than 0.1%

^d^Seq-Dupl-level, sequence duplication level

All sequencing data (fastq files) generated in the present study are available in the NCBI Sequence Read Archive (SRA) (https://www.ncbi.nlm.nih.gov/sra/) under accession number SRP110260.

**Fig. 1:**
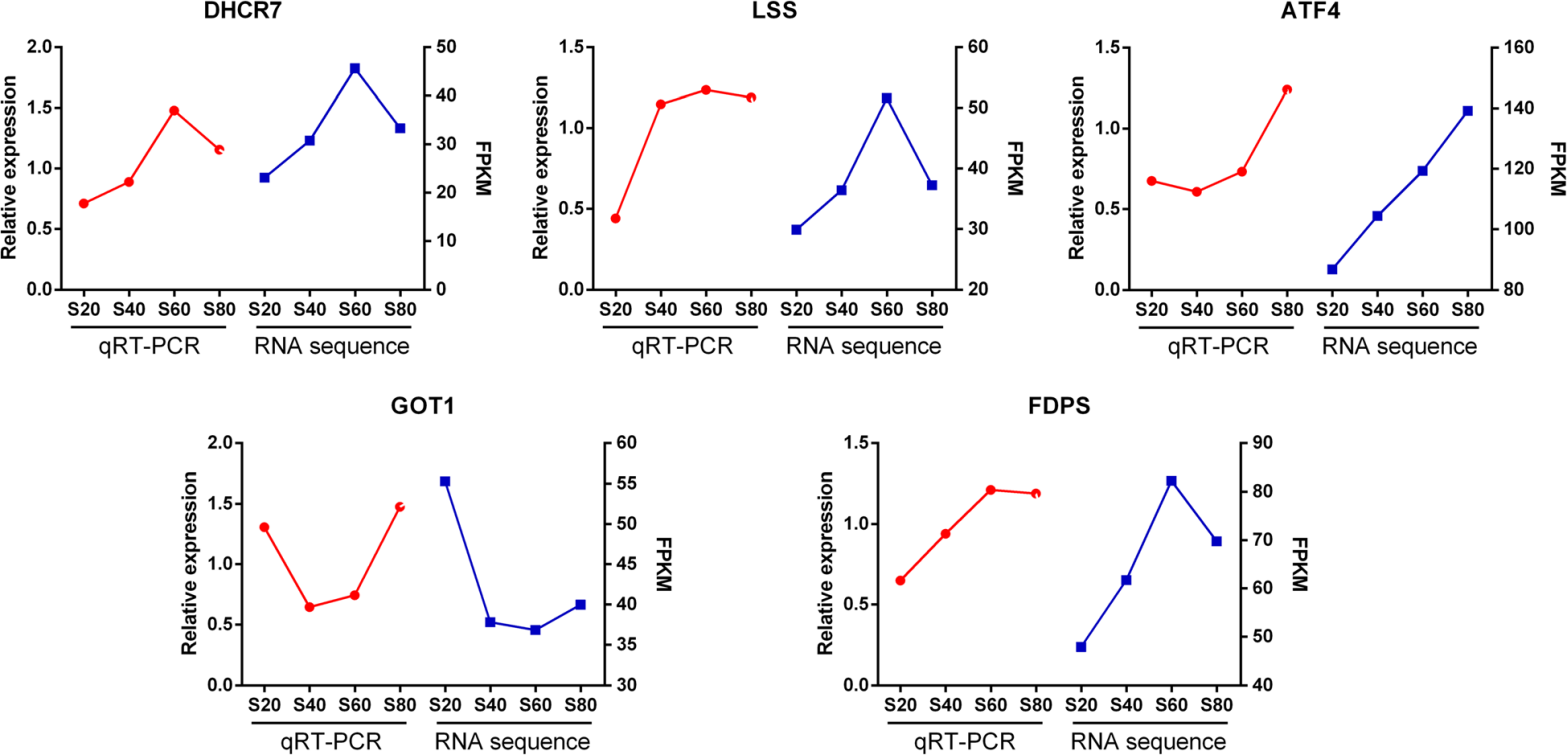
Comparison of qRT-PCR and RNA-Seq expression ratios for selected genes. Red curves represent results of qRT-PCR; blue curves represent results from RNA sequence. FPKM, fragments per kilobase of exon per million fragments mapped

When selected gene expression levels between qRT-PCR and RNA-Seq platforms were compared, a strong average correlation (r = 0.88) was observed, confirming the high reproducibility of the data. For all five genes, the fold changes among treatments in qRT-PCR were consistent with those in the RNA-Seq data (Fig. 1).

### Differential gene expression

**Fig. 2:**
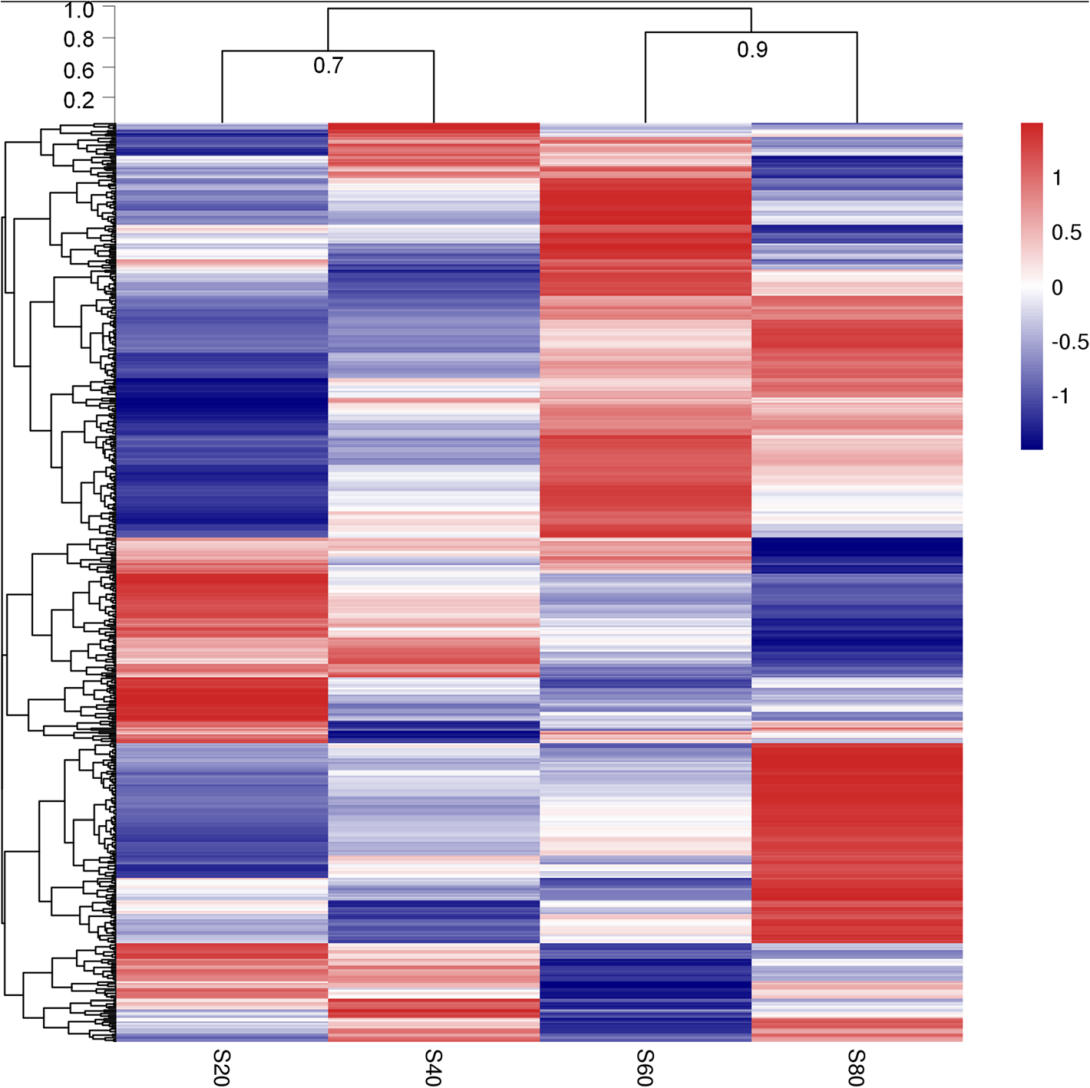
Heatmap showing hierarchical clustering of 532 differentially expressed genes among treatments. The log2 ratio values of DEG abundance were used for hierarchical cluster analysis with the R pheatmap package. Red and blue indicate relative over- or under-expression of genes, respectively. Dietary treatments were corn silage-based diets that consisted of 20%, 40%, 60%, and 80% corn silage (on a DM basis, namely the S20, S40, S60, and S80 groups)

A total of 10,005 unigenes were detected in the bovine liver. In addition, 532 DEGs were identified among the treatment groups. The number of DEGs between groups were 81 (S20 vs. S40), 225 (S20 vs. S60), 258 (S20 vs. S80), 112 (S40 vs. S60), 161 (S40 vs. S80), and 138 (S60 vs. S80). The details of the DEGs, including gene IDs, symbols, descriptions, and statistical information are shown in Additional file 2: Table S4. The heatmap of the DEGs was generated by hierarchical cluster analysis of gene expression traits (Fig. 2). The resultant heat map revealed a clear bifurcation of treatments into two broad clusters that were generally segregated into low-forage (S20 and S40, similarity = 0.7) and high-forage (S60 and S80, similarity = 0.9) dietary treatments.

### Functional analysis of all DEGs among four dietary treatments

**Fig. 3:**
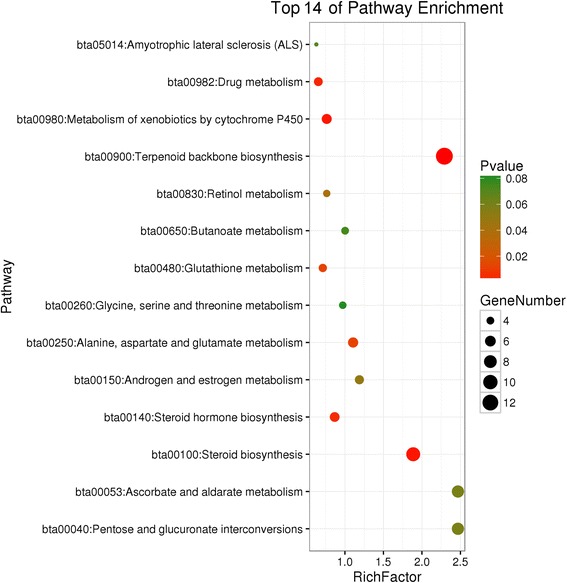
KEGG pathway enrichment analysis of differentially expressed genes (top 14 pathways listed according to *P*-value)

The GO analysis revealed that 76, 14, and eight different (*P* ≤ 0.05) terms were enriched for BP, MF, and CC, respectively (Additional file 1: Table S5). Among them, five BP-terms related to lipid metabolism, namely cholesterol biosynthetic process, sterol biosynthetic process, lipid biosynthetic process, cholesterol metabolic process, and sterol metabolic process, as well as one other BP (oxidation reduction) were significantly (*q*-value ≤0.05) enriched among the four dietary treatments (Table 5). The DEGs related to lipid metabolism were *PCK1*, *MSMO1*, *FDPS*, *IDI1*, *MVK*, *SOAT2*, *PTGS1*, *CYP11A1*, *HMGCR*, *MOGAT1*, *P2RX7*, *HMGCS1*, *MVD*, *FADS2*, *DHCR7*, *ACACA*, *LSS*, and *AGMO*. Nine significant pathways were enriched (*P* ≤ 0.05) in the KEGG analysis, most of which were related to lipid, amino acid, and carbohydrate metabolic pathways (Fig. 3).

**Table 5:** Gene Ontology analysis of differentially expressed genes (only significant terms listed)

GO ID^a^	GO Terms	Counts^b^	*P*-value	*q*-value
BP-GO:0006695	cholesterol biosynthetic process	7	4.64E-06	0.007729
BP-GO:0016126	sterol biosynthetic process	7	9.26E-06	0.015432
BP-GO:0008610	lipid biosynthetic process	17	9.88E-06	0.016469
BP-GO:0055114	oxidation reduction	32	1.31E-05	0.021806
BP-GO:0008203	cholesterol metabolic process	9	1.41E-05	0.023523
BP-GO:0016125	sterol metabolic process	9	2.79E-05	0.046546

^a^*GO* Gene Ontology

^b^Number of differentially expressed genes

### Expression pattern and functional analysis of DEGs involved in the significant gene expression profiles

**Fig. 4:**
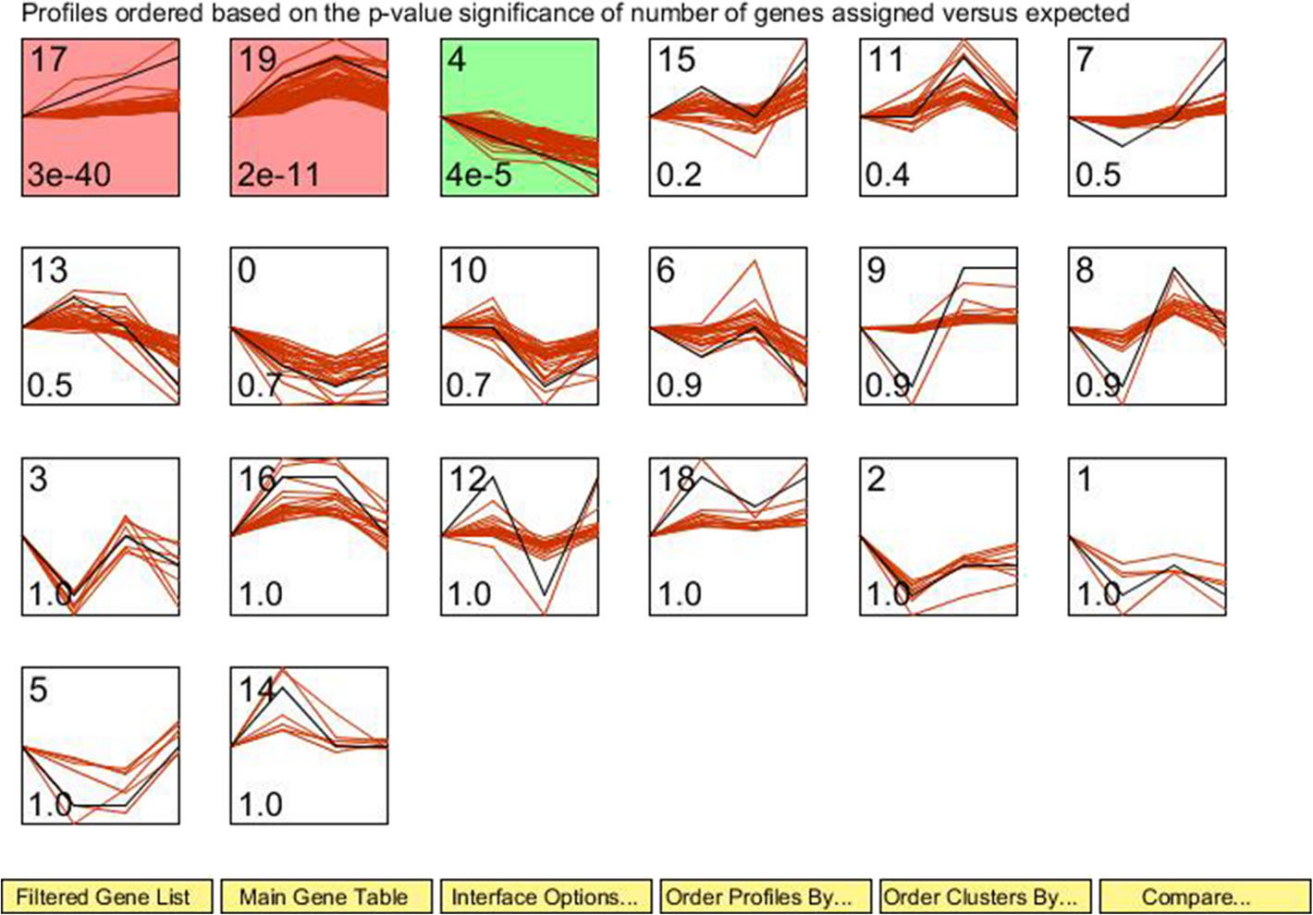
Dynamic expression pattern profiles of differentially expressed genes among treatments. Short Time-series Expression Miner (STEM) clustering analysis was performed to identify clusters; each cluster contained various numbers of DEGs with similar expression patterns. The top left-hand corner indicates the cluster ID. The lower left-hand corner contains the *P*-value of the number of assigned genes compared with the expected value. The black lines show model expression profiles. The red lines represent all individual gene expression profiles. The *x-*axis represents the dietary corn silage inclusion levels. The time series was log-normalized to start at 0. The *y-*axes of all genes in a cluster box are at the same scale

Figure 4 shows that three gene expression profiles (17, 19, and 4) were significant, among 20 candidate profiles. The abundance of DEGs in profile 17 increased monotonically with increasing dietary forage levels (112 DEGs; *P* = 3 × 10^− 40^). Expression of genes in profile 19 was upregulated when dietary forage levels increased from 20% to 60%, and then downregulated when dietary forage levels increased from 60% to 80% (73 DEGs; *P* = 2 × 10^− 11^). Expression levels of DEGs clustered in profile 4 decreased monotonically with increasing dietary forage levels (47 DEGs; *P* = 4 × 10^− 5^). The list of DEGs and their expression levels in the three profiles are shown in Additional file 1: Table S5.

**Fig. 5:**
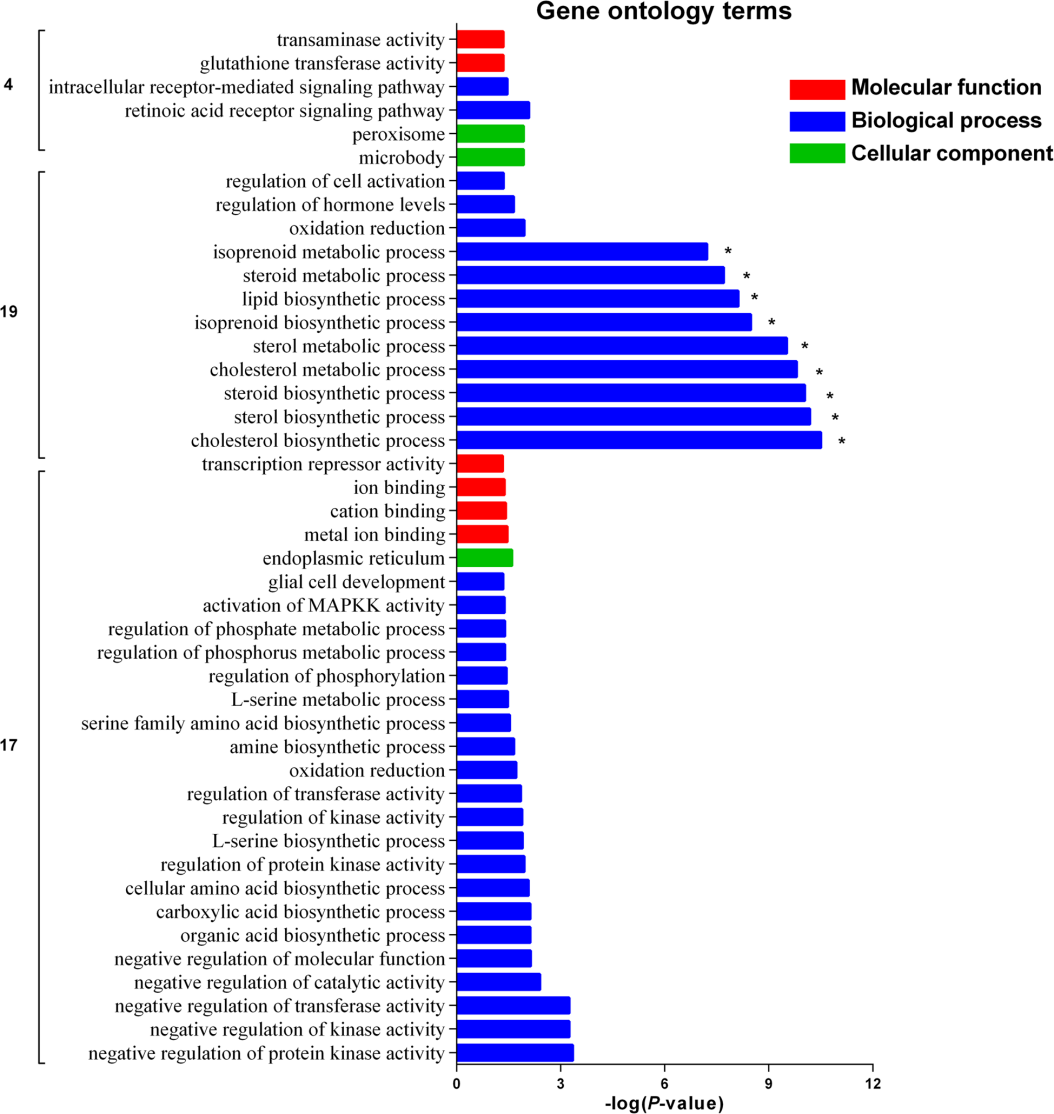
GO analysis of differentially expressed genes (*P* < 0.05) in profiles 17, 19, and 4. Red bars represent molecular function (MF) terms; blue bars represent biological process (BP) terms; green bars represent cellular component (CC) terms. Asterisks represent significantly enriched terms (FDR < 0.05). GO, Gene Ontology

Results showed that DEGs clustered in profile 17 were enriched in 26 GO categories (*P* ≤ 0.05), including 21 BP-terms, one CC-Term, and four MF-Terms (Fig. 5). Furthermore, KEGG pathway analysis showed that the p53 signaling pathway (three DEGs; *P* = 0.038) and mitogen-activated protein kinase (MAPK) signaling pathway (five DEGs; *P* = 0.038) were significantly enriched by DEGs in profile 17 (Table 6).

**Table 6:** Summary of the KEGG analysis of significant differentially expressed genes clustered in STEM

Profile	Category ID	KEGG pathway	Genes	*P*-value
17	KEGG-bta04115	p53 signaling pathway	*GADD45B, GADD45G, CDKN1A*	0.037508
	KEGG-bta04010	MAPK signaling pathway	*HSPB1, ATF4, GADD45G, GADD45B, FGF21*	0.038207
19	KEGG-bta00900	Terpenoid backbone biosynthesis	*HMGCR, HMGCS1, MVK, IDI1, MVD, FDPS*	2.68E-09
	KEGG-bta00100	Steroid biosynthesis	*DHCR7, LSS, MSMO1, SQLE*	5.75E-05

The DEGs in expression profile 19 were enriched (*P* ≤ 0.05) in 12 BP-terms and two CC-Terms (Fig. 5). Among them, the BP-terms involved in cholesterol biosynthetic process, sterol biosynthetic process, steroid biosynthetic process, cholesterol metabolic process, sterol metabolic process, isoprenoid biosynthetic process, lipid biosynthetic process, steroid metabolic process, and isoprenoid metabolic process were significantly enriched (*q*-value ≤0.05) (Fig. 5). In addition, the terpenoid backbone biosynthesis pathway (six DEGs; *P* = 2.68 × 10^− 9^) and steroid biosynthesis pathway (four DEGs; *P* = 5.75 × 10^− 5^) were significantly enriched by DEGs in profile 19 in KEGG (Table 6).

Only two BP-terms (retinoic acid receptor signaling pathway and intracellular receptor-mediated signaling pathway) and two MF-terms (glutathione transferase activity and transaminase activity) were enriched (*P* ≤ 0.05) by DEGs clustered in profile 4 (Fig. 5), and no significant KEGG pathway was enriched.

## Discussion

### Animal performance

Consistent with previous studies [8, 12], all groups had a similar intake of ME and CP, indicating that the limit-feeding model was successfully established in the present study. As the amount of feed offered was determined by the ME of the diets, the DMI was increased with increasing dietary forage levels (Additional file 1: Table S2). As less rumen degradable protein was provided, ruminal NH_3_-N concentration was reduced for the high-forage treatment compared to the low-forage treatment (*P* < 0.01, Table 1), which is consistent with the findings of previous studies [8, 12]. In ruminants, VFAs are the predominant source of energy that is absorbed from dietary sources [13]. The chemical composition of substrates could affect the molar ratios of ruminal VFAs, and the fermentation of structural carbohydrates could yield higher levels of acetate and lower levels of propionate as compared to the fermentation of starch [32]. In this study, the proportions of acetate were increased, whereas the proportions of propionate and butyrate were reduced with increasing dietary forage levels, which is consistent with the findings of other studies [9, 12]. After absorption, VFAs are mainly metabolized in PDV and the liver; acetate is mainly converted to fatty acids and propionate is mainly converted to glucose [33, 34]. The net release of acetate and propionate in the rumen accounted for about 70% and 55% of their portal net releases, respectively [34]. Previous studies suggest that propionate could be the original precursor of about 27%–54% of circulating glucose [35]. In comparison to acetate, the energy utilization efficiency of propionate was greater (52.3%–56.3% vs. 32.9%–44.4%). This apparently lower efficiency of acetate might be responsible for the depression in energy utilization of high-fiber feeds [36, 37]. The greater percentage of propionate combined with the similar levels of total volatile fatty acids (TVFAs) in the rumen indicate that an increased supply of glycogenic substrates and/or energy resources could be provided to ruminants. It also suggests that a reasonable ruminal propionate content could also improve energy metabolism efficiency [36, 38].

As a feed with a much lower neutral detergent fiber (NDF) digestibility was fed, heifers should have had increased digesta in the gastrointestinal tract, thereby leading to increased gut fill in the high-forage groups compared to in the low-forage groups [9, 39]. Consequently, the ADG and FE were greater in the low-forage groups (Table 2), which is consistent with the findings of previous studies [21, 40, 41]. As heifers had a similar intake of’ME, the differences observed in ADG and FE, as well as in VFAs composition, suggest that the overall ME (of the entire body) might be reassigned to total heat energy (including heat increment and maintenance energy) and growth energy, when different F:C diets are fed. Furthermore, these findings might suggest that the efficiency of energy utilization is greater in low-forage diets than in high-forage diets. Similarly, Reynolds et al. [6] found that 75% of forage-fed heifers had comparatively greater whole-body heat production and lower tissue energy retention. Nevertheless, it should be noted that the experimental period might not have been long enough to produce reliable estimates of FE, and the general equation used to predict gut fill might not have been suitable for the specific situations of the present study. As a result, the long-term effects of limit-feeding on growth performance, as well as improved methods to monitor the actual BW of heifers should be considered in further studies.

### DEGs and pathways clustered in profile 19

After digestion, the chemical constituents of the feed are further metabolized and subsequently transported to the liver [14]. Previous studies report that in order to utilize energy more effectively and efficiently when different F:C diets are fed, the liver has the capacity to regulate its metabolic activities, and even its size, to match whole-body energy requirements [5, 13]. In the present study, some key genes associated with lipid metabolism, particularly cholesterol and steroid metabolism, were significantly altered in the liver when subjected to different F:C diets.

**Fig. 6:**
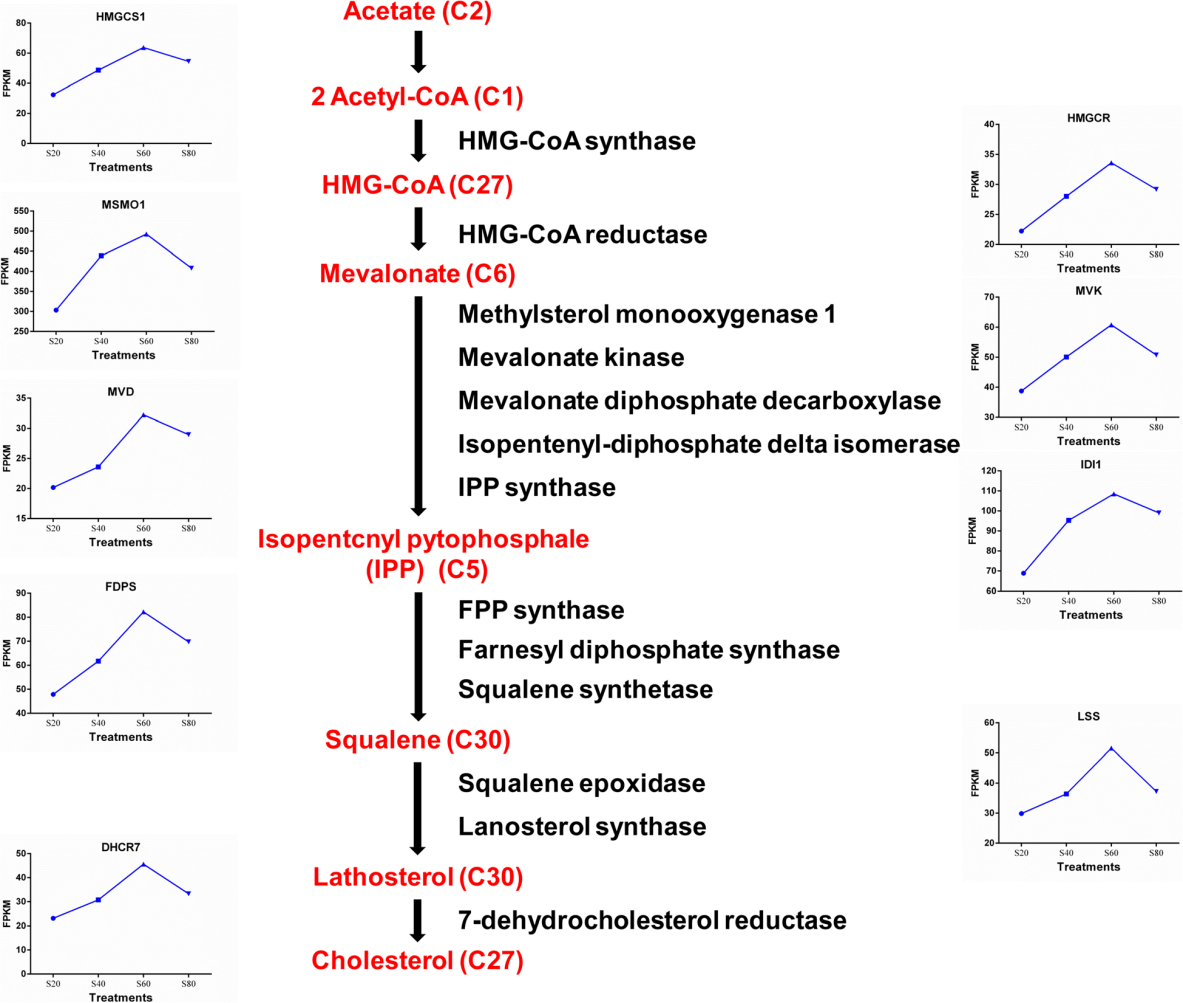
Regulation of hepatic cholesterol biosynthesis in different forage-to-concentrate ratios fed to heifers. Major metabolic intermediates are shown in red font and genes are shown in black font. Line charts represent the significant differentially expressed genes associated with cholesterol biosynthesis in this study, and mean values of FPKM are displayed. S20, 20% forage in diets; S40, 40% forage in diets; S60, 60% forage in diets; S80, 80% forage in diets. *HMGCS1*, 3-hydroxy-3-methylglutaryl-CoA; *HMGCR*, 3-hydroxy-3-methylglutaryl-CoA reductase; *MSMO1*, methylsterol monooxygenase 1; *MVK*, mevalonate kinase; *MVD*, mevalonate diphosphate decarboxylase; *IDI1*, isopentenyl-diphosphate delta isomerase 1; *FDPS*, farnesyl diphosphate synthase; *LSS*, lanosterol synthase; *DHCR7,* 7-dehydrocholesterol reductase; FPKM, fragments per kilobase of exon per million fragments mapped

Cholesterol performs a number of essential functions in the body. For example, it is a structural component of all cell membranes, thereby modulating their fluidity, and in specialized tissues, cholesterol is a precursor of bile acids, steroid hormones, and vitamin D [42]. It is critically important that the cells of the body have a steady, appropriate supply of cholesterol. The liver plays a central role in regulating cholesterol homeostasis [43]. The synthesis of cholesterol from acetyl-CoA involves more than 20 enzymatic reactions [44]. Among them, nine key genes, namely: *HMGCS1*, *HMGCR*, *MSMO1*, *MVK*, *MVD*, *IDI1*, *FDPS*, *LSS*, and *DHCR7* that encode the enzymes involved in these reactions were found to be significantly differentially expressed in the present study (Fig. 6). Key enzymes of the cholesterol biosynthetic pathway that are also associated with rate-controlling steps have been identified as HMGCR, HMGCS1, and IDI1 [31, 43, 45]. Moreover, terpenoid backbone biosynthesis (KEGG map00900) shared some steps with cholesterol biosynthesis, and was one of the precursor steps for steroid biosynthesis (KEGG map00100) in the KEGG pathway analysis. This explains the simultaneous enrichment of these two pathways in the present study [46].

All of the aforementioned genes were enriched in profile 19 and showed a quadratic increase with increasing dietary forage levels, with the S60 group yielding the highest values (Fig. 6). This indicates that cholesterol synthesis was also quadratically increased. This finding was confirmed by the TC levels in the liver (Table 3). As one of the main precursors of cholesterol, about 60%–80% of acetate is absorbed in the rumen, and protein-mediated transport pathways play a major role in its absorption [47]. The reduced cholesterol synthesis observed in the S80 group could be due to the limited absorption capacity of acetate transporter proteins when excessive acetate is produced (Table 1). Therefore, experiments that measured the activity of protein-mediated transport pathways of acetate absorption in the rumen were warranted.

In the liver, cholesterol either becomes involved in bile synthesis, or is secreted in VLDL that is delivered to the systemic circulation [48]. The transporter proteins ABCG5 and ABCG8, both of which favor the secretion of cholesterol from the liver to the bile ducts [49], were found to be upregulated in the S60 group in comparison to the S80 group (Additional file 2: Table S4). On the other hand, the plasma concentration of HDL-C and TC showed a linear increase (*P* ≤ 0.07) with increasing levels of dietary forage (Table 6). The lower concentrations of VLDL-C observed in the S60 group could be attributed to the fact that they were processed in intermediate-density lipoproteins and then metabolized to HDL.

### DEGs and pathways clustered in profile 17

The genes *HSPB1*, *ATF4*, *GADD45B*, *GADD45G*, and *FGF21* were differentially expressed and enriched in the MAPK signaling pathway in profile 17. The MAPKs both respond to extracellular stimuli (mitogens) and regulate various cellular activities [50, 51]. In addition, MAPKs also play a pivotal role in inducing steroidogenic acute regulatory protein activity, steroidogenesis, and regulating cholesterol homeostasis [46, 52]. When cows are subjected to feed restriction, cholesterol synthesis and MAPK activity are both inhibited [46]. By contrast, in the present study, both the MAPK signaling pathway and steroid biosynthesis were stimulated with increasing dietary forage levels, indicating that the MAPK signaling pathway was executing its role in regulating cholesterol homeostasis.

In this study, *GADD45B*, *GADD45G*, and *CDKN1A* were also significantly differentially expressed and enriched in the p53 signaling pathways of profile 17 (Table 4). The *GADD45* genes are important for intracellular communication in the immune system, and were found to be upregulated in the liver of cattle that had higher serum concentrations of cholesterol, or lower residual feed intake, as well as lower levels of cellular growth and proliferation, and lipid metabolism [18, 50]. The p53 signaling pathway and MAPK signaling pathway shared the same significant DEGs (*GADD45B* and *GADD45G*) in the present study. Thus, it is reasonable to infer that the changes observed in the p53 signaling pathway might be related to cholesterol biosynthesis. However, this inference should be made with caution, as only three genes were enriched in the p53 signaling pathway in the present study.

However, the synthesis of cholesterol is a highly energy-consuming process that requires 36 mol of ATP to produce 1 mol of cholesterol [53]. In addition, cholesterol metabolism reportedly has a closely relationship with whole-body energy partitioning [43, 54]. Previous studies have also reported that the liver tends to spare energy by inhibiting the biosynthesis of cholesterol to provide energy and glucose for the lactating mammary gland during feed deprivation [46, 55]. It has been suggested that lipid metabolism, especially cholesterol and sterol metabolism, can be an important mechanism to achieve energy partitioning and reassignment in cows, especially when the energy supply is required for certain physiological states, or changed by dietary composition [13, 43, 54]. Therefore, it is reasonable to infer that the increased cholesterol synthesis from acetate observed in the present study, might be one of the main reasons for the reduced efficiency of energy utilization observed in high-forage-fed heifers.

The FGF21 protein has been identified as a novel hormonal factor produced by the liver that is involved in the regulation of metabolic homeostasis and energy balance (particularly the processes of glucose and lipid metabolism) in cattle [56, 57]. Recent studies have also reported that bovine *FGF21* gene expression or circulating concentrations of FGF21 are significantly associated with BW and/or ADG and residual feed intake (RFI) rank in heifers [58, 59]. In the present study, *FGF21* mRNA expression increased with increasing dietary forage levels, and this could have occurred as a consequence of energy reassignment. This finding is consistent with the findings of previous studies that have demonstrated the response of *FGF21* to changes in energy utilization and feed intake [56, 57].

### Other important DEGs and pathways

In accordance with previous reports [18, 60, 61], our study also found that the metabolism of xenobiotics by cytochrome P450; drug metabolism; steroid hormone biosynthesis; alanine, aspartate and glutamate metabolism; glutathione metabolism; and retinol metabolism pathways (Fig. 3) were all significantly enriched in the liver and might have been involved in the regulation of feed efficiency.

Alanine and glutamine are the most glucogenic, and account for 40%–60% of the glucose formed from amino acids in ruminants [62, 63]. In this study, plasma ALT activity was increased with increasing dietary forage levels, indicating that the amount of glucose produced from alanine might be increased when heifers consume high-forage diets. This finding is in line with the changes observed in alanine, aspartate and glutamate metabolism and glutathione metabolic pathways in the liver. McCabe et al. [17] reported that *CYP11A1*, *UGT2a1*, *SULTE1*, and *CYP7A1* were altered in the steroid hormone biosynthesis pathway when cows were subjected to severe negative energy balance. In the present study, these four genes were all found to be altered in the same pathway (Additional file 2: Table S4). The *CYP7A1* gene has the ability to catalyze the rate-limiting step of the conversion of cholesterol to bile acids [17]. The *CYP11A1* gene is also known as cytochrome P450, and is closely associated with the metabolism of xenobiotics (such as drugs), and lipid homeostasis (including cholesterol, steroids, vitamin D, and bile acids) [17, 18, 60]. Tizioto et al. [61] showed that glutathione S-transferases (*MSTs*) that are associated with the catalysis of certain reactions by cytochrome P450 proteins, and the synthesis of cholesterol, steroids, and other lipids, are upregulated in inefficient cattle. These results further suggest that the efficiency of energy utilization might be reduced in high-forage-fed heifers.

## Conclusions

In this study, lower levels of ADG and FE were observed in high-forage-fed heifers, indicating that different mechanisms of energy utilization efficiency might have been involved. Hepatic cholesterol biosynthesis and steroid biosynthesis, both of which are high energy-consumption activities, as well as liver and plasma concentrations of TC, were significantly increased in heifers fed high-forage diets. The MAPK signaling pathway, which may have an important role in the regulation of steroid metabolism, also showed a linear increase with increasing dietary forage levels. Therefore, increased hepatic lipid metabolism might be responsible for the lower energy utilization efficiency observed in the heifers fed high-forage diets. This study might be useful in the future identification of individual heifers within the same dietary group that are high-efficiency phenotypes, using the upregulation of cholesterol metabolism as a proxy. In conclusion, the results of the present study provide an insight into the biology of energy utilization in heifers, and they have the potential to promote a favorable strategy to improve feed efficiency in ruminants.

## Additional files

Additional file 1: **Table S1.** Ingredient and chemical composition of experimental diets. ^1^ OM, organic matter; CP, crude protein; EE, ether extract; NDF, neutral detergent fiber; NFC, nonfiberous carbohydrate; ME, metabolizable energy. ^2^ Pre-experimental diet. ^3^ Contained 18.50% Ca; 6.00% P; 4.2% Mg; 1.4% K; 2.6% S; 7.5% Na; 12.0% Cl; 30 mg/kg of Se; 0.25% Zn; 0.25% Fe; 0.25% Mn; 1100 mg/kg Cu; 15 mg/kg I; 265,000 IU/kg vitamin A; 110,200 IU/kg vitamin D; and 2300 IU/kg vitamin E. ^4^ NFC = 100 − (NDF + CP + ether extract + ash). ^5^ Estimated as ME = total digestible nutrients × 0.04409 × 0.82, according to NRC (2001). **Table S2.** PCR primers for qRT-PCR validation of five randomly selected genes. **Table S3.** Nutrient intake of Holstein dairy heifers fed diets containing differing forage levels. **Table S5.** Gene Ontology analysis of total differentially expressed genes. **Table S6.** Differentially expressed genes clustered in Short Time-series Expression Miner (STEM) profiles 17, 19, and 4. (DOCX 71 kb)

Additional file 2: **Table S4.** Differentially expressed genes among treatments. (XLSX 76 KB)

## Abbreviations

A:P: Acetate:propionate
*ABCG5*: ATP binding cassette subfamily G member 5
*ABCG8*: ATP binding cassette subfamily G member 8
*ACACA*: Acetyl-CoA carboxylase alpha
ADG: Average daily gain
*AGMO*: Alkylglycerol monooxygenase
ALT: Alanine aminotransferase
*ATF4*: Activating transcription factor 4
BP: Biological process
BW: Body weight
CC: Cellular component
*CDKN1A*: Cyclin-dependent kinase inhibitor 1A
CP: Crude protein
*CYP11A1*: Cytochrome P450, family 11, subfamily A, polypeptide 1
DEGs: Differentially expressed genes
*DHCR7*: 7-dehydrocholesterol reductase
DM: Dry matter
DMI: Dry matter intake
ELISA: Enzyme linked immunosorbent assay
F:C: Forage:concentrate ratios
*FADS2*: Fatty acid desaturase 2
FC: Fold change
*FDPS*: Farnesyl diphosphate synthase
FE: Feed efficiency
*FGF21*: Fibroblast growth factor 21
FPKM: Fragments per kilobase of exon per million fragments mapped
*GADD45B*: Growth arrest and DNA damage inducible beta
*GADD45G*: Growth arrest and DNA damage inducible gamma
GO: Gene ontology
*GOT1*: Glutamic-oxaloacetic transaminase 1
HDL-C: High density lipoprotein cholesterol
*HMGCR*: 3-hydroxy-3-methylglutaryl-CoA reductase
*HMGCS1*: 3-hydroxy-3-methylglutaryl-CoA
*HSPB1*: Heat shock protein family B (small) member 1
*IDI1*: Isopentenyl-diphosphate delta isomerase 1
KEGG: Kyoto Encyclopedia of Genes and Genomes database
LDL-C: Low density lipoproteins cholesterol
*LSS*: Lanosterol synthase
MAPK: Mitogen-activated protein kinase
ME: Metabolizable energy
MF: Molecular function
*MOGAT1*: Monoacylglycerol O-acyltransferase 1
*MSMO1*: Methylsterol monooxygenase 1
*MVD*: Mevalonate diphosphate decarboxylase
*MVK*: Mevalonate kinase
*P2RX7*: Purinergic receptor P2X, ligand gated ion channel 7
*PCK1*: Phosphoenolpyruvate carboxykinase 1
PDV: Portal-drained viscera
*PTGS1*: Prostaglandin-endoperoxide synthase 1
SEM: Standard error of the means
*SOAT2*: Sterol O-acyltransferase 2
SRA: Sequence read archive
STEM: Short Time-series Expression Miner
TC: Total cholesterol
TG: Total triglycerides
TMR: Total mixed ration
VFAs: Volatile fatty acids
VLDL-C: Very low density lipoproteins cholesterol
