# Supplementary material for: Effects of a wide range of dietary forage-to-concentrate ratios on nutrient utilization and hepatic transcriptional profiles in limit-fed Holstein heifers
Source: BMC Genomics. 2018 Feb 17;19:148. doi: 10.1186/s12864-018-4529-9 (PMC5816523; doi:10.1186/s12864-018-4529-9)
Supplement: Supplementary file 1 — Table S1. Ingredient and chemical composition of experimental diets. 1 OM, organic matter; CP, crude protein; EE, ether extract; NDF, neutral detergent fiber; NFC, nonfiberous carbohydrate; ME, metabolizable energy. 2 Pre-experimental diet. 3 Contained 18.50% Ca; 6.00% P; 4.2% Mg; 1.4% K; 2.6% S; 7.5% Na; 12.0% Cl; 30 mg/kg of Se; 0.25% Zn; 0.25% Fe; 0.25% Mn; 1100 mg/kg Cu; 15 mg/kg I; 265,000 IU/kg vitamin A; 110,200 IU/kg vitamin D; and 2300 IU/kg vitamin E. 4 NFC = 100 − (NDF + CP + ether extract + ash). 5 Estimated as ME = total digestible nutrients × 0.04409 × 0.82, according to NRC (2001). Table S2. PCR primers for qRT-PCR validation of five randomly selected genes. Table S3. Nutrient intake of Holstein dairy heifers fed diets containing differing forage levels. Table S5. Gene Ontology analysis of total differentially expressed genes. Table S6. Differentially expressed genes clustered in Short Time-series Expression Miner (STEM) profiles 17, 19, and 4. (DOCX 71 kb) [file 12864_2018_4529_MOESM1_ESM.docx]

Table S1 Ingredient and chemical composition of experimental diets

| Item^1^ | Dietary forage levels (%) | | | | |
| --- | --- | --- | --- | --- | --- |
|  | 20 | 40 | 60 | 80 | 50^2^ |
| Ingredients, % of DM |  |  |  |  |  |
| Steam-flaked corn | 57.06 | 40.26 | 22.97 | 5.76 | 31.50 |
| Soybean meal | 18.66 | 16.05 | 13.86 | 11.39 | 15.04 |
| Corn silage | 20.00 | 40.00 | 60.00 | 80.00 | 50.00 |
| Mineral mix^3^ | 4.28 | 3.69 | 3.17 | 2.85 | 3.46 |
| Chemical composition |  |  |  |  |  |
| OM, % of DM | 92.80 | 92.96 | 93.02 | 92.89 | 92.95 |
| CP, % of DM | 15.50 | 14.60 | 13.80 | 12.90 | 14.20 |
| EE, % of DM | 3.20 | 2.90 | 2.70 | 2.40 | 2.80 |
| NDF, % of DM | 19.00 | 27.80 | 36.70 | 45.60 | 32.30 |
| NFC^4^, % of DM | 54.57 | 47.48 | 38.79 | 30.53 | 43.65 |
| ME^5^, Mcal/kg | 2.94 | 2.77 | 2.61 | 2.44 | 2.69 |
| CP:ME (g/Mcal) | 52.72 | 52.71 | 52.87 | 52.87 | 52.79 |

^1^ OM, organic matter; CP, crude protein; EE, ether extract; NDF, neutral detergent fiber; NFC, nonfiberous carbohydrate; ME, metabolizable energy.

^2^ Pre-experimental diet.

^3^ Contained 18.50% Ca, 6.00% P, 4.2% Mg, 1.4% K, 2.6% S, 7.5% Na, 12.0% Cl, 30 mg/kg of Se, 0.25% Zn, 0.25% Fe, 0.25% Mn, 1,100 mg/kg of Cu, 15 mg/kg of I, 265,000 IU/kg of vitamin A, 110,200 IU/kg of vitamin D, and 2,300IU/kg of vitamin E.

^4^ NFC = 100 – (NDF + CP + ether extract + ash).

^5^ Estimated as ME = total digestible nutrients × 0.04409 × 0.82, according to NRC (2001).

Table S2 PCR primers for qRT-PCR validation of five randomly selected genes

| Gene name | Forward primer sequence (5’-3’) | Reverse primer Sequence (5’-3’) | Amplicon (bp) | Tm (°C) |
| --- | --- | --- | --- | --- |
| *DHCR7* | GCTACACCGTCTCCACCTT | AATTCGGGGGTTAAACTCAA | 125 | 56 |
| *LSS* | GCCACCTACGAGACCAAGC | CTTCCAGCCCAAACCAG | 283 | 56 |
| *HSPB1* | AGGACGGCGTGGTGGAGAT | GGAAGGTGACGGGAATGGTG | 216 | 56 |
| *ATF4* | CCACTACTGGGTTTCCTCA | TAGCTGCTGTCTTGTTCTGC | 282 | 56 |
| *GOT1* | GGGTGACCAAGGTAAGAA | GAATCCACTACGCACAGTAA | 101 | 56 |
| *GAPDH* | CACCCTCAAGATTGTCAGC | ATAAGTCCCTCCACGATGC | 189 | 56 |
| *β-actin* | GCCATGAAGCTGAAGATGAC | CCTTCTGCAGCTCAGATATG | 229 | 56 |

Table S3 Nutrient intake of Holstein dairy heifers fed diets containing differing forage levels

| Items ^1^ | Forage levels (% of diet DM) | | | | SEM ^2^ | *P*-value | | | |
| --- | --- | --- | --- | --- | --- | --- | --- | --- | --- |
|  | 20 | 40 | 60 | 80 |  | Treatment | Linear | Quadratic | Cubic |
| DMI, kg/d | 4.42^d^ | 4.69^c^ | 4.97^b^ | 5.32^a^ | 0.107 | <0.01 | <0.01 | 0.02 | 0.50 |
| ME ^3^, Mcal/d | 12.98 | 12.98 | 12.98 | 12.98 | 0.216 | - | - | - | - |
| CP, g/d | 684.5 | 684.3 | 686.5 | 686.4 | 0.042 | - | - | - | - |
| EE, g/d | 141.3^d^ | 135.9^c^ | 134.3^b^ | 127.7^a^ | 0.200 | <0.01 | <0.01 | 0.01 | <0.01 |
| NDF, kg/d | 0.84^d^ | 1.30^c^ | 1.83^b^ | 2.43^a^ | 0.127 | <0.01 | <0.01 | 0.01 | 0.86 |
| NFC ^4^, kg/d | 2.41^a^ | 2.23^b^ | 1.93^c^ | 1.62^d^ | 0.071 | <0.01 | <0.01 | <0.01 | 0.08 |

(n = 6)

^a-b^ Means within a row with different superscripts differ (*P* < 0.05).

^1^ DMI, dry matter intake; ME, metabolizable energy; CP, crude protein; EE, ether extract; NDF, neutral detergent fiber; NFC, nonfiberous carbohydrate.

^2^ SEM, standard error of the means

^3^ Estimated as ME = total digestible nutrients × 0.04409 × 0.82, according to NRC (2001).

^4^ NFC = 100 – (NDF + CP + EE + ash).

Table S5 Gene Ontology analysis of total differentially expression genes

| GO ID | GO Term | Counts^1^ | *P*-value | FDR q value |
| --- | --- | --- | --- | --- |
| MF-GO:0016765 | transferase activity, transferring alkyl or aryl (other than methyl) groups | 8 | 1.39E-04 | 0.19884104 |
| MF-GO:0004364 | glutathione transferase activity | 6 | 1.40E-04 | 0.19995737 |
| MF-GO:0048037 | cofactor binding | 13 | 0.004964 | 6.86167373 |
| MF-GO:0030246 | carbohydrate binding | 11 | 0.005903 | 8.10946651 |
| MF-GO:0005529 | sugar binding | 7 | 0.013423 | 17.5538603 |
| MF-GO:0046983 | protein dimerization activity | 13 | 0.013576 | 17.7366504 |
| MF-GO:0019842 | vitamin binding | 8 | 0.013579 | 17.7402555 |
| MF-GO:0016564 | transcription repressor activity | 7 | 0.019118 | 24.0977037 |
| MF-GO:0016831 | carboxy-lyase activity | 4 | 0.019492 | 24.5099555 |
| MF-GO:0033549 | MAP kinase phosphatase activity | 3 | 0.032832 | 37.9254033 |
| MF-GO:0017017 | MAP kinase tyrosine/serine/threonine phosphatase activity | 3 | 0.032832 | 37.9254033 |
| MF-GO:0030170 | pyridoxal phosphate binding | 5 | 0.042798 | 46.461973 |
| MF-GO:0070279 | vitamin B6 binding | 5 | 0.042798 | 46.461973 |
| MF-GO:0005506 | iron ion binding | 12 | 0.045616 | 48.6700657 |
| CC-GO:0000267 | cell fraction | 17 | 1.77E-04 | 0.222502 |
| CC-GO:0005783 | endoplasmic reticulum | 22 | 0.002014 | 2.503148 |
| CC-GO:0005626 | insoluble fraction | 13 | 0.005037 | 6.152951 |
| CC-GO:0005625 | soluble fraction | 5 | 0.007741 | 9.30977 |
| CC-GO:0005615 | extracellular space | 14 | 0.012157 | 14.25661 |
| CC-GO:0005576 | extracellular region | 32 | 0.021552 | 23.96456 |
| CC-GO:0005624 | membrane fraction | 11 | 0.025381 | 27.62276 |
| CC-GO:0044432 | endoplasmic reticulum part | 8 | 0.041398 | 41.23602 |
| BP-GO:0006695 | cholesterol biosynthetic process | 7 | 4.64E-06 | 0.007729 |
| BP-GO:0016126 | sterol biosynthetic process | 7 | 9.26E-06 | 0.015432 |
| BP-GO:0008610 | lipid biosynthetic process | 17 | 9.88E-06 | 0.016469 |
| BP-GO:0055114 | oxidation reduction | 32 | 1.31E-05 | 0.021806 |
| BP-GO:0008203 | cholesterol metabolic process | 9 | 1.41E-05 | 0.023523 |
| BP-GO:0016125 | sterol metabolic process | 9 | 2.79E-05 | 0.046546 |
| BP-GO:0016053 | organic acid biosynthetic process | 12 | 4.37E-05 | 0.072783 |
| BP-GO:0046394 | carboxylic acid biosynthetic process | 12 | 4.37E-05 | 0.072783 |
| BP-GO:0008299 | isoprenoid biosynthetic process | 6 | 5.99E-05 | 0.099824 |
| BP-GO:0006694 | steroid biosynthetic process | 8 | 5.99E-05 | 0.099824 |
| BP-GO:0006720 | isoprenoid metabolic process | 7 | 7.45E-05 | 0.124105 |
| BP-GO:0006469 | negative regulation of protein kinase activity | 7 | 9.29E-05 | 0.154699 |
| BP-GO:0008202 | steroid metabolic process | 10 | 3.26E-04 | 0.542139 |
| BP-GO:0033673 | negative regulation of kinase activity | 7 | 3.63E-04 | 0.603176 |
| BP-GO:0051348 | negative regulation of transferase activity | 7 | 5.11E-04 | 0.84855 |
| BP-GO:0009066 | aspartate family amino acid metabolic process | 5 | 5.11E-04 | 0.84855 |
| BP-GO:0045785 | positive regulation of cell adhesion | 6 | 5.23E-04 | 0.867581 |
| BP-GO:0051338 | regulation of transferase activity | 11 | 0.001215 | 2.006154 |
| BP-GO:0008652 | cellular amino acid biosynthetic process | 6 | 0.001369 | 2.257097 |
| BP-GO:0045859 | regulation of protein kinase activity | 10 | 0.001626 | 2.67549 |
| BP-GO:0040008 | regulation of growth | 11 | 0.002688 | 4.38527 |
| BP-GO:0009309 | amine biosynthetic process | 7 | 0.003089 | 5.025256 |
| BP-GO:0043549 | regulation of kinase activity | 10 | 0.003194 | 5.190417 |
| BP-GO:0043086 | negative regulation of catalytic activity | 8 | 0.003832 | 6.196779 |
| BP-GO:0048878 | chemical homeostasis | 14 | 0.003898 | 6.300831 |
| BP-GO:0032368 | regulation of lipid transport | 4 | 0.005279 | 8.442555 |
| BP-GO:0043405 | regulation of MAP kinase activity | 6 | 0.007988 | 12.50908 |
| BP-GO:0050867 | positive regulation of cell activation | 7 | 0.008508 | 13.27099 |
| BP-GO:0050865 | regulation of cell activation | 8 | 0.009074 | 14.09192 |
| BP-GO:0019751 | polyol metabolic process | 5 | 0.009204 | 14.28001 |
| BP-GO:0009064 | glutamine family amino acid metabolic process | 5 | 0.01079 | 16.53801 |
| BP-GO:0010033 | response to organic substance | 12 | 0.012261 | 18.58173 |
| BP-GO:0044092 | negative regulation of molecular function | 8 | 0.012927 | 19.49106 |
| BP-GO:0010811 | positive regulation of cell-substrate adhesion | 4 | 1.30E-02 | 19.64121 |
| BP-GO:0045892 | negative regulation of transcription, DNA-dependent | 9 | 0.014318 | 21.36061 |
| BP-GO:0010629 | negative regulation of gene expression | 11 | 0.014959 | 22.20967 |
| BP-GO:0051253 | negative regulation of RNA metabolic process | 9 | 0.014992 | 22.25293 |
| BP-GO:0051241 | negative regulation of multicellular organismal process | 7 | 0.015231 | 22.56583 |
| BP-GO:0042325 | regulation of phosphorylation | 11 | 0.017345 | 25.2901 |
| BP-GO:0006563 | L-serine metabolic process | 3 | 0.017649 | 25.67439 |
| BP-GO:0032369 | negative regulation of lipid transport | 3 | 0.017649 | 25.67439 |
| BP-GO:0006954 | inflammatory response | 8 | 0.020379 | 29.04195 |
| BP-GO:0016481 | negative regulation of transcription | 10 | 0.022056 | 31.03982 |
| BP-GO:0034637 | cellular carbohydrate biosynthetic process | 5 | 0.022668 | 31.75498 |
| BP-GO:0019220 | regulation of phosphate metabolic process | 11 | 0.022944 | 32.07494 |
| BP-GO:0051174 | regulation of phosphorus metabolic process | 11 | 0.022944 | 32.07494 |
| BP-GO:0010817 | regulation of hormone levels | 6 | 0.023895 | 33.16885 |
| BP-GO:0002684 | positive regulation of immune system process | 9 | 0.02401 | 33.29979 |
| BP-GO:0051789 | response to protein stimulus | 5 | 0.024548 | 33.91019 |
| BP-GO:0046165 | alcohol biosynthetic process | 4 | 0.024718 | 34.10142 |
| BP-GO:0019400 | alditol metabolic process | 4 | 0.024718 | 34.10142 |
| BP-GO:0002694 | regulation of leukocyte activation | 7 | 0.025078 | 34.50549 |
| BP-GO:0030155 | regulation of cell adhesion | 6 | 0.025414 | 34.88016 |
| BP-GO:0001829 | trophectodermal cell differentiation | 3 | 0.027393 | 37.04883 |
| BP-GO:0050830 | defense response to Gram-positive bacterium | 3 | 0.027393 | 37.04883 |
| BP-GO:0009067 | aspartate family amino acid biosynthetic process | 3 | 0.027393 | 37.04883 |
| BP-GO:0009628 | response to abiotic stimulus | 9 | 0.028075 | 37.78053 |
| BP-GO:0002696 | positive regulation of leukocyte activation | 6 | 0.030335 | 40.14779 |
| BP-GO:0051094 | positive regulation of developmental process | 8 | 0.030407 | 40.22259 |
| BP-GO:0045934 | negative regulation of nucleobase, nucleoside, nucleotide and nucleic acid metabolic process | 10 | 0.03245 | 42.28668 |
| BP-GO:0042445 | hormone metabolic process | 5 | 0.032993 | 42.82393 |
| BP-GO:0051172 | negative regulation of nitrogen compound metabolic process | 10 | 0.034652 | 44.4371 |
| BP-GO:0006952 | defense response | 12 | 0.035624 | 45.36139 |
| BP-GO:0031668 | cellular response to extracellular stimulus | 4 | 0.037044 | 46.68671 |
| BP-GO:0010810 | regulation of cell-substrate adhesion | 4 | 0.037044 | 46.68671 |
| BP-GO:0044259 | multicellular organismal macromolecule metabolic process | 3 | 0.038805 | 48.28831 |
| BP-GO:0009615 | response to virus | 4 | 0.040533 | 49.81617 |
| BP-GO:0055088 | lipid homeostasis | 4 | 0.040533 | 49.81617 |
| BP-GO:0007389 | pattern specification process | 7 | 0.041959 | 51.04402 |
| BP-GO:0010605 | negative regulation of macromolecule metabolic process | 12 | 0.043621 | 52.44031 |
| BP-GO:0009266 | response to temperature stimulus | 4 | 0.044183 | 52.90391 |
| BP-GO:0043407 | negative regulation of MAP kinase activity | 3 | 0.045074 | 53.63003 |
| BP-GO:0001825 | blastocyst formation | 3 | 0.045074 | 53.63003 |
| BP-GO:0030031 | cell projection assembly | 4 | 0.04799 | 55.93439 |
| BP-GO:0051347 | positive regulation of transferase activity | 6 | 0.048514 | 56.33674 |

^1^ Numbers of differentially expressed genes.

Table S6 Differentially expressed genes clustered in Short Time-series Expression Miner (STEM) profiles 17, 19, and 4

| Profile | Gene symbol | Expression level (FPKM) | | | |
| --- | --- | --- | --- | --- | --- |
|  |  | S20 | S40 | S60 | S80 |
| 17 | A1BG | 1059.23 | 1055.22 | 1539.74 | 1654.03 |
|  | ABHD4 | 5.60 | 6.54 | 8.97 | 9.37 |
|  | AGMO | 10.27 | 13.95 | 15.84 | 16.56 |
|  | ALDH1L2 | 2.50 | 3.34 | 7.00 | 9.43 |
|  | ALKBH5 | 18.11 | 20.57 | 23.40 | 28.64 |
|  | ASPHD2 | 1.18 | 1.33 | 2.28 | 4.13 |
|  | ATF4 | 86.76 | 104.44 | 119.38 | 139.21 |
|  | ATF5 | 92.36 | 107.21 | 158.58 | 219.27 |
|  | BANP | 2.56 | 2.89 | 3.28 | 5.12 |
|  | BCL6 | 5.52 | 6.38 | 6.78 | 8.63 |
|  | C11ORF86 | 41.21 | 49.40 | 51.31 | 76.93 |
|  | C5AR1 | 1.85 | 2.10 | 2.53 | 3.39 |
|  | CACFD1 | 5.53 | 6.47 | 7.85 | 8.49 |
|  | CARD19 | 34.37 | 39.77 | 42.64 | 52.15 |
|  | CARS | 5.44 | 5.49 | 7.74 | 10.19 |
|  | CBLC | 3.63 | 4.94 | 5.59 | 5.84 |
|  | CCDC134 | 2.19 | 2.39 | 2.91 | 4.59 |
|  | CCL24 | 10.81 | 12.79 | 20.95 | 18.19 |
|  | CDK2AP2 | 14.50 | 16.68 | 22.31 | 26.71 |
|  | CDKN1A | 7.23 | 7.59 | 11.18 | 11.39 |
|  | CKAP4 | 30.06 | 37.29 | 46.17 | 46.85 |
|  | CLDN15 | 21.12 | 22.74 | 30.99 | 32.42 |
|  | COL26A1 | 0.89 | 1.06 | 2.06 | 1.77 |
|  | CRELD2 | 23.96 | 30.39 | 35.64 | 75.94 |
|  | DERL3 | 8.09 | 8.65 | 12.14 | 15.19 |
|  | DNAJB11 | 24.58 | 28.81 | 29.69 | 41.80 |
|  | DNAJC12 | 26.72 | 34.45 | 36.61 | 49.40 |
|  | EIF2B2 | 12.66 | 13.88 | 16.34 | 19.09 |
|  | EXTL1 | 8.54 | 11.71 | 13.98 | 17.32 |
|  | FAM101A | 17.96 | 21.52 | 26.91 | 43.96 |
|  | FAM43A | 1.88 | 2.24 | 2.55 | 3.59 |
|  | FGF21 | 0 | 0.94 | 1.39 | 12.60 |
|  | FNDC3B | 18.78 | 23.14 | 22.92 | 33.64 |
|  | FOXA2 | 8.57 | 8.83 | 13.85 | 16.30 |
|  | GADD45B | 12.55 | 13.56 | 15.40 | 18.97 |
|  | GADD45G | 8.18 | 14.96 | 49.83 | 39.42 |
|  | GAS1 | 4.50 | 5.17 | 7.91 | 9.14 |
|  | GMEB2 | 3.80 | 4.29 | 5.36 | 5.74 |
|  | GMPPA | 10.93 | 10.91 | 15.08 | 17.13 |
|  | GPRC5C | 36.44 | 42.36 | 46.09 | 56.13 |
|  | HAMP | 262.24 | 405.05 | 394.60 | 519.62 |
|  | HSPB1 | 476.73 | 520.11 | 534.41 | 743.22 |
|  | HSPB8 | 24.13 | 25.26 | 31.81 | 38.12 |
|  | IGDCC4 | 1.79 | 1.91 | 2.22 | 2.76 |
|  | KANK2 | 11.60 | 15.25 | 17.19 | 18.86 |
|  | KCNT2 | 1.59 | 1.88 | 2.04 | 2.53 |
|  | KIFC3 | 11.06 | 13.04 | 15.22 | 17.87 |
|  | KLHL21 | 5.74 | 6.27 | 8.31 | 9.98 |
|  | KLHL25 | 11.75 | 13.21 | 14.74 | 20.49 |
|  | LOXL4 | 3.89 | 4.73 | 6.37 | 7.35 |
|  | LSM10 | 14.53 | 17.31 | 20.91 | 23.62 |
|  | MANF | 71.77 | 93.20 | 92.29 | 155.24 |
|  | MAP1LC3A | 40.68 | 48.73 | 58.95 | 73.43 |
|  | MARS | 7.94 | 9.25 | 12.42 | 12.80 |
|  | MICAL2 | 6.56 | 8.33 | 8.34 | 13.10 |
|  | MIEF2 | 1.78 | 2.54 | 3.43 | 3.42 |
|  | MRPS26 | 23.62 | 26.99 | 35.96 | 34.63 |
|  | NOM1 | 2.82 | 3.17 | 4.40 | 4.04 |
|  | NUAK1 | 2.74 | 3.19 | 4.15 | 4.15 |
|  | PHGDH | 29.24 | 32.97 | 36.02 | 47.45 |
|  | PIM1 | 8.37 | 8.99 | 12.11 | 16.06 |
|  | PM20D2 | 7.07 | 7.40 | 9.35 | 11.75 |
|  | PPP1R12C | 4.06 | 4.68 | 5.72 | 6.30 |
|  | PRRC1 | 19.03 | 21.75 | 22.75 | 28.89 |
|  | PSPH | 2.60 | 4.59 | 6.94 | 11.35 |
|  | PYCR1 | 3.15 | 4.73 | 6.27 | 9.45 |
|  | RAB20 | 4.91 | 5.81 | 7.92 | 11.70 |
|  | RAB3D | 10.60 | 13.32 | 13.75 | 16.21 |
|  | RASIP1 | 4.28 | 5.11 | 5.75 | 6.89 |
|  | RGS16 | 5.10 | 5.32 | 6.83 | 8.82 |
|  | RTN2 | 1.86 | 1.98 | 2.28 | 3.15 |
|  | SDF2L1 | 57.93 | 74.48 | 120.86 | 132.84 |
|  | SELK | 24.96 | 28.49 | 30.39 | 40.51 |
|  | SH3BP4 | 1.69 | 2.21 | 2.47 | 2.57 |
|  | SLC16A5 | 4.10 | 4.06 | 7.21 | 8.03 |
|  | SLC19A1 | 1.46 | 1.55 | 2.55 | 2.61 |
|  | SLC25A47 | 55.18 | 52.23 | 82.42 | 104.39 |
|  | SPATA20 | 20.04 | 21.40 | 33.32 | 32.54 |
|  | SRXN1 | 3.78 | 4.07 | 5.13 | 6.31 |
|  | STEAP4 | 25.61 | 30.83 | 37.15 | 39.98 |
|  | SYT4 | 7.15 | 7.95 | 10.04 | 12.78 |
|  | SYVN1 | 19.94 | 20.78 | 24.60 | 31.33 |
|  | TEAD3 | 3.93 | 4.15 | 6.01 | 5.48 |
|  | TIMM17A | 6.92 | 7.40 | 10.58 | 12.65 |
|  | TKT | 24.71 | 30.13 | 40.49 | 41.06 |
|  | TMEM127 | 19.62 | 24.75 | 29.31 | 31.10 |
|  | TMEM14A | 15.76 | 23.53 | 23.92 | 27.44 |
|  | TMEM201 | 0.98 | 1.28 | 1.32 | 2.30 |
|  | TMEM218 | 5.00 | 6.69 | 6.35 | 8.61 |
|  | TRIB3 | 2.39 | 2.52 | 3.00 | 3.64 |
|  | TRIM7 | 3.29 | 3.74 | 5.76 | 5.73 |
|  | UGDH | 188.97 | 209.16 | 240.25 | 294.81 |
|  | WFS1 | 7.28 | 9.97 | 12.18 | 19.26 |
|  | ZBTB16 | 12.25 | 14.37 | 21.41 | 22.63 |
|  | ZFYVE21 | 49.78 | 58.38 | 66.88 | 75.19 |
|  | ZNF70 | 1.76 | 1.86 | 3.22 | 4.43 |
|  | ZXDC | 3.76 | 4.90 | 5.24 | 5.85 |
|  | BLVRB | 89.71 | 100.14 | 127.81 | 145.02 |
|  | OSGIN1 | 45.21 | 51.10 | 80.79 | 90.37 |
|  | MT1E | 1019.22 | 1651.34 | 1715.85 | 2044.07 |
|  | LOC508916 | 315.07 | 356.46 | 551.51 | 503.79 |
|  | ENSBTAG00000003492 | 43.13 | 54.78 | 62.94 | 86.20 |
|  | DMAP1 | 6.07 | 7.81 | 7.71 | 9.92 |
|  | CGN1 | 118.77 | 135.55 | 193.59 | 191.99 |
|  | MT2A | 1425.42 | 2183.86 | 2265.06 | 2966.92 |
|  | CXCL3 | 43.91 | 66.95 | 110.57 | 115.35 |
|  | MT1A | 5572.58 | 7838.94 | 8596.78 | 11882.40 |
|  | LOC516108 | 16.12 | 19.02 | 22.42 | 24.80 |
|  | ENSBTAG00000038706 | 126.65 | 175.42 | 176.57 | 275.69 |
|  | LOC615303 | 94.60 | 111.35 | 164.62 | 186.24 |
|  | ENSBTAG00000045822 | 26.83 | 34.47 | 41.78 | 56.52 |
|  | LOC789955 | 1.09 | 1.80 | 2.11 | 2.65 |
| 19 | ACOT8 | 11.64 | 13.49 | 19.21 | 15.35 |
|  | ACSS2 | 13.27 | 23.39 | 32.79 | 19.92 |
|  | ADORA3 | 2.35 | 3.08 | 5.11 | 4.15 |
|  | AIF1L | 47.53 | 70.07 | 78.95 | 61.77 |
|  | ASMTL | 21.67 | 23.95 | 36.35 | 31.50 |
|  | AXIN2 | 1.69 | 1.95 | 3.20 | 2.63 |
|  | BAIAP2 | 4.29 | 5.19 | 6.51 | 6.17 |
|  | CAPN11 | 1.76 | 2.23 | 2.73 | 1.96 |
|  | CCDC124 | 12.56 | 15.01 | 18.96 | 17.57 |
|  | CCDC17 | 1.63 | 2.44 | 2.93 | 2.10 |
|  | CCDC64 | 1.88 | 2.98 | 4.12 | 3.36 |
|  | CCS | 17.57 | 24.75 | 34.01 | 24.79 |
|  | CDAN1 | 2.59 | 3.27 | 4.37 | 3.08 |
|  | CDK9 | 32.16 | 56.69 | 84.37 | 78.25 |
|  | CISH | 3.50 | 4.43 | 6.03 | 4.17 |
|  | CLEC14A | 6.70 | 7.68 | 10.10 | 7.93 |
|  | CSAD | 12.72 | 25.89 | 37.99 | 30.31 |
|  | CUX2 | 3.01 | 4.73 | 7.30 | 5.18 |
|  | CYP11A1 | 13.58 | 20.98 | 36.77 | 22.14 |
|  | DHCR7 | 23.11 | 30.77 | 45.66 | 33.31 |
|  | EFNA4 | 7.16 | 9.93 | 10.92 | 10.33 |
|  | ELK1 | 4.98 | 7.14 | 7.94 | 7.53 |
|  | FABP1 | 1347.98 | 1497.50 | 2057.00 | 1778.03 |
|  | FAM193A | 3.85 | 4.71 | 5.84 | 4.84 |
|  | FAM207A | 4.73 | 5.76 | 8.37 | 7.30 |
|  | FETUB | 57.87 | 80.80 | 105.90 | 87.47 |
|  | FKBP11 | 59.57 | 70.77 | 94.06 | 76.47 |
|  | FXN | 3.73 | 5.78 | 5.28 | 4.79 |
|  | GIPC1 | 5.22 | 7.35 | 8.40 | 7.12 |
|  | HAPLN3 | 15.69 | 20.67 | 28.81 | 23.41 |
|  | HMGCR | 22.24 | 28.03 | 33.61 | 29.19 |
|  | HMGCS1 | 32.44 | 48.71 | 63.78 | 54.47 |
|  | IDI1 | 68.89 | 95.31 | 108.54 | 99.00 |
|  | IL27RA | 9.51 | 11.83 | 15.13 | 11.65 |
|  | ITFG2 | 4.16 | 6.19 | 6.54 | 6.62 |
|  | KIAA1462 | 2.64 | 3.75 | 4.25 | 3.59 |
|  | KLHL17 | 2.58 | 3.48 | 3.98 | 3.71 |
|  | LSS | 29.89 | 36.41 | 51.60 | 37.24 |
|  | MBOAT2 | 15.15 | 24.29 | 31.44 | 21.11 |
|  | MSMO1 | 303.42 | 438.81 | 492.55 | 408.09 |
|  | MVD | 20.17 | 23.61 | 32.24 | 28.97 |
|  | MVK | 38.75 | 50.09 | 60.78 | 50.76 |
|  | NEIL2 | 1.24 | 1.86 | 2.39 | 1.66 |
|  | NRTN | 9.93 | 14.46 | 16.60 | 14.19 |
|  | NUDT14 | 10.01 | 13.16 | 17.53 | 15.02 |
|  | OPLAH | 4.15 | 5.36 | 6.34 | 6.22 |
|  | PCSK1N | 1.16 | 2.40 | 3.45 | 3.04 |
|  | PLD4 | 2.58 | 4.71 | 4.36 | 4.31 |
|  | RAB26 | 1.08 | 2.21 | 3.00 | 2.14 |
|  | RDH11 | 13.39 | 16.80 | 21.43 | 16.99 |
|  | RNF43 | 3.18 | 4.72 | 4.86 | 4.42 |
|  | SCN1B | 2.27 | 4.57 | 4.38 | 4.03 |
|  | SELM | 3.39 | 5.59 | 8.92 | 5.48 |
|  | SELP | 4.37 | 5.90 | 6.92 | 5.92 |
|  | SLC9A1 | 1.88 | 2.35 | 3.01 | 2.68 |
|  | SQLE | 28.06 | 41.19 | 49.51 | 44.66 |
|  | THAP4 | 5.19 | 6.78 | 9.02 | 6.88 |
|  | TLE2 | 3.93 | 4.80 | 6.04 | 4.79 |
|  | TMEM258 | 71.06 | 75.76 | 111.70 | 94.67 |
|  | TNIP2 | 4.11 | 5.32 | 6.72 | 5.29 |
|  | ZMAT5 | 6.77 | 7.53 | 12.20 | 9.49 |
|  | MRPL23 | 330.26 | 413.80 | 529.45 | 383.21 |
|  | ENSBTAG00000003367 | 4.28 | 7.71 | 11.32 | 7.74 |
|  | FDPS | 47.91 | 61.73 | 82.28 | 69.75 |
|  | MZT2B | 19.17 | 25.11 | 34.11 | 27.59 |
|  | ENSBTAG00000020620 | 1.65 | 4.31 | 6.81 | 4.26 |
|  | BORCS8 | 12.69 | 14.65 | 19.58 | 16.01 |
|  | ENSBTAG00000035144 | 5.48 | 6.88 | 11.45 | 9.95 |
|  | LOC518623 | 23.94 | 47.49 | 68.78 | 64.25 |
|  | ENSBTAG00000039928 | 15.35 | 27.07 | 43.33 | 38.83 |
|  | ENSBTAG00000040334 | 6.96 | 8.71 | 10.80 | 9.41 |
|  | ENSBTAG00000045728 | 47.39 | 64.04 | 106.50 | 63.32 |
|  | ENSBTAG00000047113 | 8.52 | 12.45 | 17.50 | 14.58 |
| 4 | ABAT | 39.55 | 34.27 | 30.02 | 25.38 |
|  | AMDHD1 | 62.17 | 47.97 | 43.37 | 37.20 |
|  | ARHGEF37 | 3.38 | 2.46 | 2.53 | 1.75 |
|  | B3GALT5 | 8.15 | 5.58 | 4.70 | 4.23 |
|  | BCAS1 | 6.41 | 4.25 | 3.74 | 3.28 |
|  | CLDN4 | 8.64 | 8.49 | 6.93 | 4.87 |
|  | CYP26A1 | 5.92 | 4.94 | 3.85 | 1.99 |
|  | CYP2U1 | 24.08 | 20.92 | 20.15 | 14.67 |
|  | DNASE2B | 5.71 | 5.55 | 3.81 | 3.29 |
|  | DPY19L3 | 4.16 | 3.46 | 2.74 | 2.51 |
|  | DUSP10 | 8.89 | 8.71 | 5.83 | 5.83 |
|  | EML6 | 2.97 | 3.18 | 1.97 | 1.84 |
|  | FERMT1 | 3.17 | 2.38 | 2.17 | 1.71 |
|  | FIBIN | 2.26 | 1.93 | 1.57 | 1.19 |
|  | FKBP5 | 6.57 | 5.68 | 4.50 | 4.16 |
|  | GLS2 | 31.05 | 21.26 | 18.49 | 15.80 |
|  | GPR37 | 3.87 | 3.08 | 2.99 | 2.32 |
|  | GSDMB | 26.33 | 21.82 | 14.88 | 13.78 |
|  | GSTM1 | 147.34 | 125.16 | 114.59 | 91.39 |
|  | KMO | 43.37 | 34.19 | 24.97 | 19.34 |
|  | MOGAT1 | 27.01 | 22.11 | 16.05 | 14.22 |
|  | NAV3 | 2.68 | 2.27 | 1.94 | 1.78 |
|  | NT5DC1 | 11.10 | 8.83 | 7.50 | 6.01 |
|  | NUGGC | 15.24 | 13.70 | 11.38 | 7.98 |
|  | OAT | 16.00 | 14.32 | 7.96 | 7.42 |
|  | PLS1 | 10.06 | 10.98 | 7.97 | 7.03 |
|  | RARB | 4.82 | 2.69 | 2.62 | 2.09 |
|  | SESN3 | 9.34 | 7.66 | 7.40 | 4.66 |
|  | SHE | 7.90 | 6.25 | 4.80 | 4.96 |
|  | SLC16A12 | 23.87 | 23.00 | 17.31 | 15.39 |
|  | SLC18B1 | 2.91 | 2.50 | 1.66 | 1.74 |
|  | SMARCA1 | 23.38 | 20.75 | 19.12 | 14.91 |
|  | SULT1E1 | 52.79 | 26.31 | 24.98 | 14.55 |
|  | SWAP70 | 46.56 | 43.41 | 30.05 | 27.15 |
|  | TRPM8 | 19.36 | 15.14 | 13.26 | 11.49 |
|  | TYW3 | 8.41 | 6.90 | 5.84 | 5.50 |
|  | UGT2A3 | 46.87 | 41.29 | 35.49 | 30.74 |
|  | WASF3 | 13.66 | 10.40 | 8.29 | 6.06 |
|  | WEE1 | 7.85 | 6.38 | 5.61 | 4.78 |
|  | WIF1 | 2.73 | 1.92 | 1.89 | 1.44 |
|  | XPNPEP2 | 10.69 | 6.59 | 5.64 | 5.14 |
|  | ZNF484 | 4.14 | 3.48 | 2.81 | 2.65 |
|  | MTMR9 | 55.56 | 49.72 | 42.62 | 24.59 |
|  | GSTM2 | 244.13 | 174.13 | 152.47 | 123.67 |
|  | LOC516355 | 2.13 | 1.45 | 1.32 | 1.18 |
|  | B3GNT2 | 14.41 | 12.16 | 9.83 | 8.53 |
|  | LOC100300442 | 46.98 | 42.68 | 31.47 | 20.26 |
